# Bioinformatics design of a peptide vaccine containing sarcoma antigen NY-SAR-35 epitopes against breast cancer and evaluation of its immunological function in BALB/c mouse model

**DOI:** 10.1371/journal.pone.0306117

**Published:** 2024-06-26

**Authors:** Nour Samman, Hassan Mohabatkar, Mandana Behbahani, Mazdak Ganjlikhani Hakemi

**Affiliations:** 1 Department of Biotechnology, Faculty of Biological Science and Technology, University of Isfahan, Isfahan, Iran; 2 Department of Immunology, Faculty of Medicine, Isfahan University of Medical Sciences, Isfahan, Iran; 3 Regenerative and Restorative Medicine Research Center (REMER), Research Institute for Health Sciences and Technologies (SABITA), Istanbul Medipol University, Istanbul, Turkey; 8th Medical Center of Chinese PLA General Hospital, CHINA

## Abstract

The development of a cancer vaccine has become an essential focus in the field of medical biotechnology and immunology. In our study, the NY-SAR-35 cancer/testis antigen was targeted to design a novel peptide vaccine using bioinformatics tools, and BALB/c mice were used to evaluate the vaccine’s immunological function. This evaluation involved assessing peptide-specific IgG levels in the serum via ELISA and measuring the levels of IFN-γ, IL-4, and granzyme B in the supernatant of cultured splenocytes. The final vaccine construct consisted of two T lymphocyte epitopes linked by the AAY linker. This construct displayed high antigenicity, non-allergenicity, non-toxicity, stability, and ability to induce IFN-γ and IL-4. It showed stable dynamics with both human MHC-I and II molecules, as well as mouse MHC-II molecules, and revealed strong Van der Waals and electrostatic energies. Emulsifying our peptide vaccine in incomplete Freund’s adjuvant resulted in a remarkable increase in the levels of IgG. The splenocytes of mice that received the combination of peptide and adjuvant displayed a noteworthy increase in IFN-γ, IL-4, and granzyme B secretion. Additionally, their lymphocytes exhibited higher proliferation rates compared to the control group. Our data demonstrated that our vaccine could stimulate a robust immune response, making it a promising candidate for cancer prevention. However, clinical trials are necessary to assess its efficacy in humans.

## Introduction

Breast cancer is the most prevalent form of cancer among women, exerting a large influence on the global number of cancer deaths [1]. Malignant transformation of normal mammary cells leads to the development of breast tumors, characterized by uncontrolled growth, proliferation, and invasion of cells [2]. Despite the use of radiotherapy, chemotherapy, and surgery for breast cancer treatment, patient prognosis remains unsatisfactory, particularly in those with metastatic disease [3]. Furthermore, the effectiveness of these therapies is limited by their side effects. As a result, there has been a growing interest in immunotherapy, specifically cancer vaccines, as a potential treatment option for breast cancer. These therapies offer tumor specificity, favorable patient tolerance, safety, and the ability to establish long-term immune memory, aiding in the prevention of tumor recurrence and relapse [4].

Cancer vaccines can be classified into three categories: peptide or protein-based vaccines, vector-based vaccines, and cellular-based vaccines [5]. Peptide-based cancer vaccines can induce a cell-mediated immune response against tumors by presenting tumor antigen epitopes to T cells. Once the T cells are activated, they can identify and react to the tumor antigens presented on the surface of cancer cells, initiating an immune response that ultimately results in the elimination of cancer cells by T cells [6].

There are two types of tumor antigens: tumor-specific antigens (TSAs) and tumor-associated antigens (TAAs). TSAs consist of neoantigens produced by mutated cancer genes and those derived from oncoviruses, while TAAs are grouped into three categories: overexpressed antigens, differentiation antigens, and cancer/testis antigens (CTAs). CTAs are uniquely expressed in immune-privileged sites and can thus evade immune recognition [7]. The high immunogenicity and tissue-specific expression of CTAs highlight their potential role as cancer biomarkers and treatment targets. The expression of CTAs has been observed in various tumor types, while in normal tissues, their expression remains limited to the testis and placenta. This restricted expression, combined with their potent immunogenic properties, makes them highly promising targets for immunotherapy [8]. Sarcoma antigen NY-SAR-35, also known as FMR1NB or CT37, was identified using the serological analysis of recombinant cDNA expression (SEREX) method. It is a protein comprising 255 amino acids, and its coding is determined by a gene located on the human X chromosome. Methylation of its promoter region may regulate its expression [9]. It has been identified as a positive regulator of mitosis in tumor cells [10], with a function in regulating microtubule nucleation and, or anchoring events in the mitotic spindle. Its aberrant expression has been observed in several cancers, such as melanoma, sarcoma, lung, breast, bladder, esophageal, and ovarian cancer tissues. Moreover, it has been detected in various cancer cell lines, including those of sarcoma, multiple myeloma, chronic myeloid leukemia, lung cancer, and breast cancer (such as the BT20 breast cancer cell line, which lacks both estrogen and progesterone receptors). The expression of NY-SAR-35 has been shown to stimulate cellular growth, proliferation, and invasiveness in both SNU-449 (a cell line derived from hepatocellular carcinoma) and SK-LC-14 (a cell line derived from lung cancer) [9, 11, 12]. As a prospective target for immunotherapy, NY-SAR-35 holds considerable promise due to its potential to stimulate an immune response against cancer cells [13].

In cancer vaccine formulations, adjuvants are often included and administered alongside tumor antigens to enhance their delivery and effectiveness [14]. Adjuvants are generally classified into two groups: immunostimulants, such as toll-like receptor agonists, and those that enable the deposition of antigens, such as emulsions. Emulsions are subcategorized as either water-in-oil (W/O) or oil-in-water (O/W) formulations, and their primary function is to act as a carrier for antigens at the injection site, enabling a gradual and sustained release of immunogenic agents. Incomplete Freund’s adjuvant (IFA), a water-in-oil (W/O) emulsion without heat-killed mycobacteria, is the most well-known adjuvant in laboratory research [15]. It is recognized as the gold standard within this category of adjuvants for evaluating the antigenicity of antigens in murine models. The human equivalent, Montanide ISA-51, finds extensive use in formulations for peptide cancer vaccines. It has been utilized in several trials, including melanoma and renal carcinoma [16].

Determining dominant epitopes experimentally and assessing vaccine efficacy can be a costly and time-consuming procedure. Therefore, bioinformatics approaches have gained popularity as alternative methods for vaccine design. These methods aid researchers in designing vaccines by identifying T and B-cell epitopes from studied antigens and predicting their binding ability to MHC molecules, thereby stimulating specific immune responses. A common approach for vaccine design through bioinformatics involves three steps: epitope prediction, vaccine engineering, and vaccine evaluation [17].

In the present study, the NY-SAR-35 antigen was chosen for designing a peptide cancer vaccine. The prediction of major histocompatibility complex class I and II (MHC-I and MHC-II)-restricted epitopes was conducted using bioinformatics tools. To identify the most promising epitopes, various parameters, including antigenicity, allergenicity, toxicity, stability, and the capacity to elicit interferon-gamma, were considered. Two epitopes with the highest score of antigenicity, non-allergenicity, non-toxicity, and stability, as well as the ability to stimulate the production of both IFN-γ and IL-4, were selected and linked together using a suitable linker for the development of our final vaccine construct. Various properties and the structure of the designed construct were predicted. Furthermore, the binding affinity of the vaccine to MHC molecules was assessed by molecular docking, and the stability of the resulting complexes was examined using molecular dynamics simulations. Then, the capability of our designed vaccine to evoke an immune response through *in vivo* experiments was evaluated, marking the first instance of such a vaccine. These experiments involved determining the levels of IgG antibodies, measuring the levels of specific cytokines, and assessing the cytotoxicity activity by measuring the level of the granzyme B in BALB/c mice.

## Materials and methods

### Bioinformatics

#### Protein sequence retrieval

The reference amino acid sequence of NY-SAR-35 was retrieved from the UniProt database (www.uniprot.com) and used as an input for epitope prediction by bioinformatics tools. The corresponding accession number for this sequence is Q8N0W7.

#### Prediction of MHC-I and MHC-II binding epitopes

NetMHCpan BA 4.1 method on IEDB (http://tools.iedb.org/mhci/) was utilized to predict the MHC-I binding epitopes. It employs artificial neural networks (ANNs) to predict peptide binding to various MHC-I molecules with known sequences [18]. The MHC-I alleles (*H-2-Ld*, *H-2-Kd*, *H-2-Dd*, *H-2-Qa1*, and *H-2-Qa2*) expressed in BALB/c mice were selected. The strong binding threshold was defined as % Rank ≤ 1. Furthermore, The NetMHCIIpan BA 4.1 method on IEDB (http://tools.iedb.org/mhcii/) was applied to predict MHC-II binding epitopes.

It utilizes ANNs to predict peptide binding to MHC-II molecules with known sequences [19]. The MHC-II alleles (*H2-IAd* and *H2-IEd*) expressed in BALB/c mice were chosen. The strong binding threshold was set at % Rank ≤ 2. Additionally, the same approach was applied to predict human MHC-I and MHC-II binding epitopes. A panel comprising 16 HLA-A alleles (A*01:*01*, A*26:*01*, A*32:*01*, A*02:*01*, A*02:*03*, A*02:*06*, A*68:*02*, A*23:*01*, A*24:*02*, A*03:*01*, A*11:*01*, A*30:*01*, A*31:*01*, A*33:*01*, A*68:*01*, and A*30:*02*) and 11 HLA-B alleles (B*40:*01*, B*44:*02*, B*44:*03*, B*57:*01*, B*58:*01*, B*15:*01*, B*07:*02*, B*35:*01*, B*51:*01*, B*53:*01*, and B*08:*01*) were chosen. These alleles collectively represent 97% of HLA-A and B allelic variants in most ethnic populations. The set of MHC-II alleles (HLA-DRB1*01:*01*, HLA-DRB1*03:*01*, HLA-DRB1*04:*01*, HLA-DRB1*04:*05*, HLA-DRB1*07:*01*, HLA-DRB1*08:*02*, HLA-DRB1*09:*01*, HLA-DRB1*11:*01*, HLA-DRB1*12:*01*, HLA-DRB1*13:*02*, HLA-DRB1*15:*01*, HLA-DRB3*01:*01*, HLA-DRB3*02:*02*, HLA-DRB4*01:*01*, HLA-DRB5*01:*01*, HLA-DQA1*05:01/DQB1*02:*01*, HLA-DQA1*05:01/DQB1*03:*01*, HLA-DQA1*03:01/DQB1*03:*02*, HLA-DQA1*04:01/DQB1*04:*02*, HLA-DQA1*01:01/DQB1*05:*01*, HLA-DQA1*01:02/DQB1*06:*02*, HLA-DPA1*02:01/DPB1*01:*01*, HLA-DPA1*01:03/DPB1*02:*01*, HLA-DPA1*01:03/DPB1*04:*01*, HLA-DPA1*03:01/DPB1*04:*02*, HLA-DPA1*02:01/DPB1*05:*01*, and HLA-DPA1*02:01/DPB1*14:*01*) capable of providing over 99% population coverage across all major ethnicities worldwide were selected [20, 21]. Predicted MHC-I-restricted epitopes in mice were compared with MHC-I-restricted epitopes in humans to identify the shared epitopes. The same way was applied to MHC-II binding epitopes. After identifying the shared epitopes, their cores were selected for further screening. The core peptide resides within the grooves of the MHC molecule and serves as the primary component in forming peptide-MHC (pMHC) complexes.

#### Antigenicity analysis

The antigenicity of the predicted epitopes was evaluated by VaxiJen v2.0 at threshold = 0.5 (http://www.ddg-pharmfac.net/vaxijen/VaxiJen/VaxiJen.html). It represents a novel alignment-free bioinformatics tool for the *in silico* identification of antigens, relying on the auto cross covariance (ACC) transformation of protein sequences into uniform vectors that capture principal amino acid properties [22].

#### Allergenicity and toxicity analysis

Potential allergenicity and toxicity of the predicted epitopes were estimated using AllergenFP v.1.0 (https://ddg-pharmfac.net/AllergenFP/index.html) and ToxinPred (https://webs.iiitd.edu.in/raghava/toxinpred/) web servers. AllergenFP v.1.0 employs a novel alignment-free descriptor-based fingerprint approach to discern allergens from non-allergens. This method is done within a four-step algorithm. Initially, protein sequences are characterized based on amino acid principal properties. Subsequently, the resulting strings of varying lengths are standardized into vectors of equal length through ACC. These vectors are then translated into binary fingerprints and compared using the Tanimoto coefficient [23]. ToxinPred utilizes a support vector machine (SVM) to predict the toxicity. Identification of potential motifs in toxins relies on meme (Multiple Em for Motif Elicitation) and motif alignment & search tool (MAST). By integrating motif identification with SVM output, the hybrid approach provides an additional benefit, enhancing the biological reliability of toxic peptide prediction [24]. This server enables toxicity prediction for peptides containing fewer than 50 amino acids [25].

#### Stability prediction

The stability of the predicted epitopes was assessed via ProtParam (https://web.expasy.org/protparam/). If an epitope has an instability index of less than 40, it is expected to be stable. Conversely, if the instability index is greater than 40, the epitope may be unstable [26].

#### Prediction of cytokine production

IFNepitope (http://crdd.osdd.net/raghava/ifnepitope/) was used to predict the MHC-II-restricted epitopes with a capacity to activate IFN-γ cytokine production. It is based on two models: Motif based and SVM-based models [27]. IL4pred (https://webs.iiitd.edu.in/raghava/il4pred/), an SVM-based predictor, was used to predict IL-4 inducing MHC-II binders [28].

#### The final construct of the peptide vaccine

The antigenic epitopes were selected based on their non-allergenicity, non-toxicity, stability, and ability to induce IFN-γ and IL-4. These epitopes were linked using various amino acid linkers, including either Gly-Pro-Gly-Pro-Gly (GPGPG) or Ala-Ala-Tyr (AAY). Finally, the construct with the highest antigenicity score, as determined by VaxiJen v2.0, was chosen.

#### The constructed vaccine features

The allergenicity of the final construct selected from the previous step was predicted by AllerTOP v.2.0 (https://www.ddg-pharmfac.net/AllerTOP/), wherein a machine learning technique employing k-nearest neighbors is utilized for the classification of allergens and non-allergens [29]. The toxicity of the final construct was predicted by ToxinPred. Furthermore, the ability to induce IFN-γ and IL-4 were assessed using IFNepitope and IL4pred, respectively. Several physicochemical properties and hydrophobicity/hydrophilicity of the final construct were evaluated via ProtParam, PepCalc (https://pepcalc.com/), and Peptide2 (https://www.peptide2.com/N_peptide_hydrophobicity_hydrophilicity.php). Protein-Sol (https://protein-sol.manchester.ac.uk/) was employed to assess the solubility of the constructed vaccine. Utilizing protein solubility data from *E*. *coli*, Protein-Sol predicts protein solubility. According to the experimental dataset’s population average (PopAvrSol) of 0.45, a solubility value exceeding 0.45 indicates higher solubility compared to the average *E*. *coli* protein [30].

#### Secondary and tertiary structure prediction of the constructed vaccine

The secondary structure of the constructed vaccine was predicted using Prabi through the SOPMA secondary structure prediction method available at (https://npsa-prabi.ibcp.fr/cgibin/npsa_automat.pl?page=/NPSA/npsa_sopma.html). This approach involves several steps: (1) the creation of a restricted database of protein sequences, alongside their established secondary structures; (2) the use of a similarity algorithm to predict the secondary structure of all proteins within the database; (3) the identification of prediction parameters that optimize the accuracy of the prediction; and (4) application of these parameters to the designated protein [31]. In addition, the 3D structure of the vaccine construct was produced by PEPFOLD3 (http://bioserv.rpbs.univ-paris-diderot.fr/services/PEP-FOLD3). PEPFOLD3 is a *de novo* method to predict peptide structures based on amino acid sequences (5–50 amino acids). This server typically delivers solutions within few minutes due to its speedy performance [32].

#### Quality assessment of predicted 3D structure

The geometry quality of the predicted 3D structure was assessed based on the Ramachandran plot using the PROCHECK tool in SAVESv6.0-Structure Validation Server (https://saves.mbi.ucla.edu/). This tool checks the stereochemical quality of a protein structure by examining the geometry of individual residues as well as the overall structure geometry [33]. ERRAT tool in SAVESv6.0 was utilized to confirm the 3D structure of the predicted model. It examines the nonbonded interactions between various types of atoms and generates a plot that displays the error function value relative to the position of a sliding window consisting of nine residues. This calculation is based on a comparison with statistics obtained from highly refined structures [34]. Additionally, the model quality was further confirmed using ProSA-web (https://prosa.services.came.sbg.ac.at/prosa.php), a widely employed tool for identifying potential errors in 3D models of protein structures [35].

#### Identification of the conformational B cell epitopes in the 3D structure of the constructed vaccine

Considering the crucial role of B cells in anti-tumor humoral immune responses, conformational B cell epitopes in the constructed vaccine were predicted using the ElliPro tool (http://tools.iedb.org/ellipro/). The 3D structure of the vaccine construct was utilized as an input for ElliPro. This tool utilizes a modified version of Thornton’s method, residue clustering algorithms, the MODELLER program, and the Jmol viewer [36].

#### Molecular docking analysis

Peptide-protein molecular docking was employed to assess the binding affinity of the constructed vaccine to mouse MHC-I and MHC-II molecules. PDB files of MHC alleles were retrieved from the RCSB database (https://www.rcsb.org). Mouse MHC alleles, including *H-2Kd* (PDB entry 5GSV), *H-2-Ld* (PDB entry 1LDP), *H-2-Dd* (PDB entry 5IVX), and *H-2-IAd* (PDB entry 2IAD) were used for peptide-mouse MHC docking analyses. In addition, the constructed vaccine was docked with the most commonly expressed alleles in humans, HLA-A*02:*01* (MHC-I) and HLA-DRB1*01:*01* (MHC-II). The corresponding PDB entries for these structures are 4UQ3 and 1AQD, respectively. The molecular docking was done using HPEPDOCK 2.0 (http://huanglab.phys.hust.edu.cn/hpepdock/), a tool that performs flexible peptide-protein docking by rapid modeling of peptide conformations and comprehensive sampling of binding orientations [37].

#### Molecular Dynamics Simulation (MDS)

The molecular dynamics simulation was employed to understand the dynamic behavior and mode of binding for the docked complexes. Eight systems were examined, each consisting of one of the following components: Human MHC-I, Human MHC-II, Mouse MHC-I, and Mouse MHC-II, either in their free states or complex with a vaccine. Simulations were conducted using the GROMACS 2022.6 package [38], employing the Amber99SB force field. The initial structures for the molecular dynamic simulations were obtained from the best docking poses. The systems were solvated in a cubic water box using the tip3p model. To maintain system charge neutrality and achieve a physiologically relevant ionic strength of 0.15 M, sufficient amounts of Na^+^ and Cl^-^ ions were added. The simulations were carried out in four steps. The first step involved removing additional forces acting on the atoms and conducting energy minimization for relaxation. In the second and third steps, 1 nanosecond simulations were performed using the canonical (NVT) and isothermal-isobaric (NPT) ensembles to maintain a constant temperature and pressure. Heavy atoms were restrained. In the final step, position restraints were removed, and 200 ns simulations were run with a time step of 2 femtoseconds. Trajectories were recorded for further analysis. Energy minimization in the first step used the steepest descent method [39]. The Nose-Hoover thermostat [40] and Parinello-Rahmann barostat [41] were employed in the subsequent stages to maintain temperature and pressure. The Lennard-Jones potential, particle mesh Ewald method [42], and LINCS algorithm [43] were utilized for Van der Waals interactions, long-range electrostatic interactions, and covalent bond constraints. Various properties of the systems were analyzed, including root mean square deviation (RMSD), root mean square fluctuation (RMSF), radius of gyration (Rg), solvent accessible surface area (SASA), and hydrogen bonds. The binding energy of the vaccine with the MHCs was determined using the molecular mechanics Poisson–Boltzmann surface area (MM-PBSA) method [44].

#### Immune simulation of the constructed vaccine

To predict probable immune responses of the constructed vaccine, computational immune simulation was performed via C-ImmSim (https://kraken.iac.rm.cnr.it/C-IMMSIM/). It employs a position-specific scoring algorithm (PSSM) to predict the interaction of immunogenic epitopes with the immune system [45].

### Peptide synthesis

The constructed peptide vaccine was synthesized by TAG Copenhagen A/S, Denmark. The composition and purity of the construct were verified by mass spectroscopy and HPLC analysis. The vaccine was determined to be 88.01% pure. The peptide was dissolved in phosphate-buffered saline (PBS) (pH 7.4) with 5% dimethyl sulfoxide (DMSO).

### In vivo studies

#### Animal care and handling

Female BALB/c mice weighing approximately 22 ± 2 g were procured from the Pasteur Institute of Tehran, Iran. They were provided with unrestricted access to standard laboratory chow and water throughout the study. The laboratory conditions were carefully maintained at a relative humidity of 50 ± 10%, a temperature of 24 ± 2 ºC, and a light/dark cycle of 12 hours each.

#### Mice immunization

The mice used in our study were given a minimum of two weeks to acclimate before being randomly divided into four groups (n = 21). The first group (PBS) received a subcutaneous injection of 100 μl of PBS and served as a negative control (n = 3). The second group (Adjuvant) was injected subcutaneously with 100 μl of incomplete Freund’s adjuvant (Sigma, US) formulated with PBS in a 50% V/V ratio (n = 6). The third group (Peptide) received a subcutaneous injection of 100 μl of peptide (100 μg peptide in 100 μl of PBS with 5% DMSO) (n = 6), while the fourth group (Adj+pep) received a subcutaneous injection of a mixture of 50 μl peptide plus 50 μl incomplete Freund’s adjuvant (n = 6). The mice were given three injections spaced two weeks apart (on days 0, 14, and 28). Blood samples and splenocytes were collected two weeks after the second injection (Adjuvant 2nd, Peptide 2nd, and Adj+pep 2nd), and two weeks after the third injection (Adjuvant 3rd, Peptide 3rd, and Adj+pep 3rd) [46].

#### Antibodies determination by ELISA

Two weeks after the second injection, half of the mice from each group were sacrificed using an overdose of ketamine and xylazine. Similarly, two weeks after the third injection, the remaining half of the mice from each group were sacrificed, and their blood was collected. An indirect homemade ELISA assay was utilized to determine the serum levels of peptide-specific IgG antibodies. In summary, a 96-well microtiter plate (Maxisorb, Costar, USA) was coated with 20 μg/ml peptide in 0.1 M carbonate buffer (pH 9.6) (200 μL) overnight at 4°C. The wells were then washed three times using 0.05% Tween 20 in PBS (PBS-T). Afterward, the blocking solution, containing 1% bovine serum albumin (BSA), was added to the wells. The plate was then incubated for 1 hour at 37°C. After washing the wells twice with PBS, sera were added (three wells per sample), and the plate was further incubated for 2 hours at 37°C. To serve as the secondary antibody, HRP-conjugated goat anti-mouse IgG antibody (Biolegend, San Diego-California) was used. Subsequently, 3,3′,5,5′-tetramethylbenzidine (TMB) substrate was added. Following 15 minutes of incubation in the dark, the reaction was stopped using 100 μl of 2 M sulfuric acid, and the absorbance was measured at 490 nm via an ELISA reader (Bio-Rad 680, USA) [46].

#### Cytokine production and granzyme B secretion assay

Half of the immunized mice from each group were sacrificed two weeks after receiving their second injection. Similarly, the remaining half of the mice from each group were sacrificed two weeks after the third injection—the method employed for euthanasia involved utilizing a ketamine/xylazine overdose. The spleens were collected under completely sterile conditions, and splenocytes were cultured on a 24-well cell culture plate. The culture medium employed was RPMI 1640 (Sigma, USA) supplemented with 10% fetal bovine serum (FBS) (Sigma, USA) and a 1% mixture of antibiotics containing penicillin (Sigma, USA) and streptomycin (Sigma, USA). The cultured splenocytes were then re-stimulated with 5 mg/mL of peptide and incubated at 37 ℃ with 5% CO2 for 48 hours. Following this, the levels of IFN-γ and IL-4 cytokines in the supernatant of the wells were measured using commercial ELISA kits (Karmania Pars Gene, Iran), according to the manufacturer’s instructions. Additionally, granzyme B levels were measured in the supernatant of wells utilizing an ELISA kit manufactured by ZellBio GmbH in Germany, following the provided instructions [47].

#### Antigen‑specific proliferation assay

The proliferation of lymphocytes was assessed using the 3-(4,5-dimethylthiazol-2-yl)-2,5-diphenyl tetrazolium bromide (MTT) assay. The cultured splenocytes were seeded into a 96-well plate (8×10^5^ cells per well). Afterward, the peptide was added to the wells (2 μg peptide/well) to stimulate the splenocytes. To serve as negative controls, some wells were not treated with the peptide. The assay relies on the enzymatic activity of mitochondrial dehydrogenases in splenocytes, which convert the water-soluble yellow substrate MTT into a dark blue insoluble formazan product. A solubilization solution (DMSO) was next added to dissolve the previously formed insoluble purple formazan, resulting in a colored solution. The absorbance of each well was read at a wavelength of 570 nm using a microplate ELISA reader. The results were presented as a stimulation index, calculated as the ratio of the mean absorbance of the peptide-stimulated cells to that of unstimulated cells [47].

#### Behavioral investigations

To assess the safety of the vaccine, the mice were closely monitored throughout the experiment for changes in appearance, and alterations in behavioral patterns such as weakness, aggressiveness, refusal of food or water, and signs of pain or illness. Additionally, their body weight was measured daily to track any changes.

### Ethics statement

All animal procedures for this study were approved by the Biomedical Research Ethics Committees University of Isfahan, Isfahan, Iran (Ethics committee reference number: IR.UI.REC.1402.027). All methods were performed under the relevant guidelines and regulations for the care and use of laboratory animals. The study is reported under ARRIVE guidelines.

### Statistical analysis

Multiple comparisons of one-way ANOVA were performed using GraphPad Prism 10.1.2 software to examine differences between the analyzed groups, employing Tukey’s post hoc tests. The statistical significance was determined by setting a threshold of P < 0.05.

## Results

### Bioinformatics

#### MHC-I and MHC-II-restricted epitopes prediction

The amino acid sequence of NY-SAR-35 was submitted to IEDB for the prediction of MHC-I and MHC-II-restricted epitopes. A total of 27 MHC-I-restricted epitopes (Table 1) and 3 MHC-II-restricted epitopes (Table 2) were shared between mice and humans.

**Table 1:** Shared MHC-I-restricted epitopes, their cores, and alleles.

MHC-I-restricted epitopes	Core sequence	Mouse allele	Human allele
FSSSGTTSF	**FSSSGTTSF**	*H-2-Qa1*	HLA-B*35:*01* HLA-B*15:*01* HLA-B*53:*01* HLA-B*58:*01* HLA-A*26:*01* HLA-A*01:*01*
LPIYCRSLF	**LPIYCRSLF**	*H-2-Ld*	HLA-B*07:*02* HLA-B*51:*01* HLA-B*35:*01* HLA-B*53:*01*
SENAHGQSL	**SENAHGQSL**	*H-2-Qa2*	HLA-B*40:*01* HLA-B*44:*02* HLA-B*44:*03*
VSKPFGMLM	**VSKPFGMLM**	*H-2-Qa1*, *H-2-Dd*, *H-2-Ld*	HLA-B*57:*01* HLA-A*30:*01* HLA-B*58:*01*
TSFKCFAPF	**TSFKCFAPF**	*H-2-Qa1*, *H-2-Dd*, *H-2-Ld*, *H-2-Qa2*	HLA-A*24:*02* HLA-B*53:*01* HLA-A*30:*02* HLA-B*15:*01* HLA-A*32:*01* HLA-B*57:*01* HLA-B*58:*01* HLA-A*23:*01* HLA-A*26:*01*
VPKQMMQMF	**VPKQMMQMF**	*H-2-Ld*	HLA-B*53:*01* HLA-B*51:*01* HLA-B*35:*01* HLA-B*07:*02*
MQMFGLGAI	**MQMFGLGAI**	*H-2-Qa2*, *H-2-Kd*, *H-2-Qa1*	HLA-B*08:*01* HLA-A*02:*06* HLA-A*02:*03* HLA-A*32:*01* HLA-B*40:*01* HLA-B*15:*01* HLA-A*02:*01*
YLCSGSSYF	**YLCSGSSYF**	*H-2-Qa1*	HLA-B*15:*01* HLA-B*35:*01* HLA-A*24:*02* HLA-A*23:*01* HLA-A*32:*01* HLA-A*26:*01*
RVSKPFGML	**RVSKPFGML**	*H-2-Qa1*	HLA-A*32:*01* HLA-B*07:*02* HLA-A*30:*01*
LEALLNFFF	**LEALLNFFF**	*H-2-Qa2*	HLA-B*44:*03* HLA-B*44:*02* HLA-B*40:*01*
YFVLANGHI	**YFVLANGHI**	*H-2-Kd*	HLA-A*23:*01* HLA-A*24:*02*
KQMMQMFGL	**KQMMQMFGL**	*H-2-Qa2*, *H-2-Qa1*, *H-2-Kd*	HLA-A*02:*06* HLA-A*32:*01* HLA-A*02:*01* HLA-B*15:*01* HLA-B*40:*01* HLA-A*30:*02*
FGMLMLSIW	**FGMLMLSIW**	*H-2-Dd*	HLA-B*53:*01* HLA-B*58:*01* HLA-B*57:*01*
FFFPTTCNL	**FFFPTTCNL**	*H-2-Kd*	HLA-A*23:*01* HLA-A*24:*02*
SALEALLNF	**SALEALLNF**	*H-2-Qa1*, *H-2-Ld*	HLA-A*23:*01* HLA-B*51:*01* HLA-B*35:*01* HLA-B*58:*01* HLA-B*53:*01* HLA-A*32:*01* HLA-B*57:*01*
AMRVAHLEL	**AMRVAHLEL**	*H-2-Qa1*, *H-2-Ld*	HLA-A*30:*01* HLA-B*08:*01* HLA-B*07:*02* HLA-B*15:*01* HLA-A*32:*01*
YYLCSGSSY	**YYLCSGSSY**	*H-2-Kd*	HLA-A*30:*02* HLA-B*35:*01* HLA-A*24:*02* HLA-A*23:*01*
FGLGAISLI	**FGLGAISLI**	*H-2-Dd*, *H-2-Kd*	HLA-B*51:*01*
CLPIYCRSL	**CLPIYCRSL**	*H-2-Dd*	HLA-B*08:*01*
MLSIWILLF	**MLSIWILLF**	*H-2-Qa1*	HLA-A*32:*01* HLA-A*23:*01* HLA-A*24:*02* HLA-B*15:*01* HLA-B*53:*01*
MADRPQPGW	**MADRPQPGW**	*H-2-Qa1*	HLA-B*57:*01* HLA-B*58:*01* HLA-B*53:*01*
LMLSIWILL	**LMLSIWILL**	*H-2-Ld*	HLA-A*02:*01* HLA-A*32:*01* HLA-A*02:*06* HLA-A*23:*01*
VAHLELATY	**VAHLELATY**	*H-2-Qa1*	HLA-B*15:*01* HLA-A*30:*02* HLA-B*35:*01*
FVLANGHIL	**FVLANGHIL**	*H-2-Qa1*	HLA-A*02:*06* HLA-B*35:*01*
EEDSALEAL	**EEDSALEAL**	*H-2-Qa2*	HLA-B*40:*01*
RPQPGWRES	**RPQPGWRES**	*H-2-Ld*	HLA-B*07:*02*
FVCYYLSYY	**FVCYYLSYY**	*H-2-Qa1*	HLA-B*35:*01* HLA-A*30:*02* HLA-B*15:*01* HLA-A*26:*01* HLA-A*01:*01*

**Table 2:** Shared MHC-II-restricted epitopes, their cores, and alleles.

MHC-II-restricted epitopes	Core sequence	Mouse allele	Human allele
RRSHRAMRVAHLELA	**HRAMRVAHL**	*H2-IAd*	HLA-DQA1*04:01/DQB1*04:*02*
SHRAMRVAHLELATY	**MRVAHLELA**	*H2-IAd*	HLA-DRB4*01:*01* HLA-DQA1*01:02/DQB1*06:*02* HLA-DPA1*02:01/DPB1*14:*01*
SSYFVLANGHILPNS	**FVLANGHIL**	*H2-IEd*	HLA-DRB1*01:*01* HLA-DRB1*09:*01* HLA-DRB1*07:*01* HLA-DRB1*13:*02* HLA-DRB5*01:*01* HLA-DRB3*02:*02* HLA-DRB1*15:*01*

#### Antigenicity analysis

Seven of the shared MHC-I-restricted epitopes had a VaxiJen score higher than 0.5, and two of the shared MHC-II-restricted epitopes were found to be antigenic.

#### Allergenicity and toxicity analysis

Ten of the shared MHC-I-restricted epitopes and one of the shared MHC-II-restricted epitopes were found to be non-allergen. All of the MHC-I and MHC-II restricted epitopes were non-toxic.

#### Stability prediction

Sixteen of the shared MHC-I-restricted epitopes and two of the shared MHC-II-restricted epitopes were predicted to be stable.

#### Prediction of cytokine production

All shared MHC-II-restricted epitopes displayed a positive IFNepitope score, indicating their ability to stimulate the production of IFN-γ. Moreover, they were predicted to be IL4-inducers.

Tables 3 and 4 provide results regarding antigenicity, allergenicity, toxicity, stability, and the ability to stimulate cytokines for shared MHC-I and MHC-II-restricted epitopes.

**Table 3:** The properties of shared MHC-I-restricted epitopes.

MHC-I-restricted epitopes	VaxiJen score/ result	AllergenFP v.1.0 result	ToxinPred result	Instability index/ result	Final decision
FSSSGTTSF	0.1237/non antigenic	allergen	non-toxin	42.26/unstable	-
LPIYCRSLF	-0.1460/non antigenic	allergen	non-toxin	79.11/unstable	-
SENAHGQSL	0.4491/non antigenic	allergen	non-toxin	58.16/unstable	-
VSKPFGMLM	0.1566/non antigenic	non-allergen	non-toxin	21.91/stable	-
TSFKCFAPF	0.2962/non antigenic	allergen	non-toxin	34.99/stable	-
VPKQMMQMF	-0.2132/non antigenic	non-allergen	non-toxin	45.02/unstable	-
MQMFGLGAI	-0.4132/non antigenic	allergen	non-toxin	-8.92/stable	-
YLCSGSSYF	0.4580/non antigenic	non-allergen	non-toxin	30.29/stable	-
RVSKPFGML	0.0793/non antigenic	allergen	non-toxin	21.91/stable	-
LEALLNFFF	0.7527/antigenic	allergen	non-toxin	-7.81/stable	-
YFVLANGHI	0.3245/non antigenic	allergen	non-toxin	41.01/unstable	-
KQMMQMFGL	-0.1773/non antigenic	allergen	non-toxin	23.62/stable	-
FGMLMLSIW	-0.8742/non antigenic	allergen	non-toxin	8.89/stable	-
FFFPTTCNL	1.3594/antigenic	allergen	non-toxin	30.29/stable	-
SALEALLNF	0.8274/antigenic	allergen	non-toxin	-7.81/stable	-
AMRVAHLEL	0.7354/antigenic	allergen	non-toxin	-8.92/stable	-
YYLCSGSSY	0.8706/ antigenic	non-allergen	non-toxin	44.00/unstable	-
FGLGAISLI	-1.5696/non antigenic	non-allergen	non-toxin	-0.54/stable	-
CLPIYCRSL	0.2627/non antigenic	non-allergen	non-toxin	100.51/unstable	-
MLSIWILLF	-0.2396/non antigenic	allergen	non-toxin	30.29/stable	-
MADRPQPGW	-0.6303/non antigenic	non-allergen	non-toxin	82.70/unstable	-
LMLSIWILL	-0.6058/non antigenic	allergen	non-toxin	30.29/stable	-
VAHLELATY	0.2382/non antigenic	allergen	non-toxin	-0.54/stable	-
EEDSALEAL	0.8469/antigenic	non-allergen	non-toxin	87.91/unstable	-
RPQPGWRES	-0.5164/non antigenic	non-allergen	non-toxin	108.20/unstable	-
**FVCYYLSYY**	0.7614/antigenic	non-allergen	non-toxin	36.31/stable	*

**Table 4:** The properties of shared MHC-II-restricted epitopes.

MHC-II-restricted epitopes	VaxiJen score/ result	AllergenFP v.1.0 result	ToxinPred result	Instability index/ result	IFNepitope score	IL4pred score/result	Final decision
**HRAMRVAHL**	1.4497/antigenic	non-allergen	non-toxin	-8.92/ stable	+ 0.065420626	0.30/IL4-inducer	*
MRVAHLELA	0.6876/antigenic	allergen	non-toxin	-8.92/stable	+ 0.1526113	0.33/IL4-inducer	-
FVLANGHIL	-0.0154/non antigenic	allergen	non-toxin	62.41/ unstable	+ 0.067899391	0.29/IL4-inducer	-

FVCYYLSYY and HRAMRVAHL epitopes were chosen as the final epitopes. Among the MHC-I-restricted epitopes, the FVCYYLSYY epitope was selected due to its antigenic nature, lack of allergenicity, non-toxic properties, and stability. The HRAMRVAHL epitope was selected from the MHC-II-restricted epitopes due to its notable antigenicity, lack of allergenicity, non-toxicity, and high stability. Furthermore, it demonstrated the ability to induce IFN-γ and IL-4.

#### The final construct of the peptide vaccine

**Fig 1:**
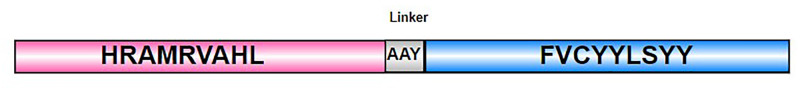
Graphical presentation of the constructed vaccine (Vac1). The vaccine included an MHC-II-restricted epitope (pink region) and an MHC-I-restricted epitope (blue region) that were merged by the AAY linker (grey region).

The selected FVCYYLSYY and HRAMRVAHL epitopes were joined together to construct the peptide vaccine. This construction utilized either the AAY linker to enhance epitope presentation by antigen-presenting cells (APCs) to immune cells and reduce junctional immunogenicity or the GPGPG linker, which plays an essential function in immune processing by limiting the formation of "junctional epitopes". The Vac1, developed using linked epitopes by AAY, demonstrated an antigenicity score of 0.8402, while the Vac2, developed using linked epitopes by GPGPG, displayed an antigenicity score of 0.7036. Therefore, based on the highest antigenicity score, Vac1 was considered the final construct (Fig 1).

#### The constructed vaccine features

The results of AllerTOP and ToxinPred showed that the Vac1 was non-allergenic and non-toxic. The IFNepitope score of +1.6218624 revealed that Vac1 could induce IFN-γ, and the IL4pred score of 0.21 indicated its capability to induce IL-4 production. The results of ProtParam showed that the Vac1 was stable and exhibited thermal stability. The grand average of hydropathicity (GRAVY) calculated was 0.367, indicating the hydrophobic nature of Vac1. The results of Peptide2 revealed that the constructed vaccine consists of 47.62% hydrophobic residues, 0% acidic residues, 19.05% basic residues, and 33.33% neutral residues. The Protein-Sol server predicted a scaled solubility value of 0.541 at a 0.45 threshold, indicating a higher solubility than the average soluble *E*. *coli* protein. Furthermore, other properties have been included in Table 5.

**Table 5:** The physicochemical characteristics of the constructed vaccine (Vac1) using ProtParam, Peptide2, and PepCalc.

Property	Measurement	Indication
Instability index	13.64	Stable
Aliphatic index	83.81	Thermostable
The grand average of hydropathicity (GRAVY)	0.367	Hydrophobic
Half-life	*in vitro* mammalian reticulocyte 3.5 hours *in vivo* yeast 10 min *E*. *coli >* 10 hours	*-*
Hydrophobicity/hydrophilicity analysis	47.62% hydrophobic residues 0% acidic residues 19.05% basic residues 33.33% neutral residues	*-*
Molecular weight	2598.01 g/mol	*-*
Isoelectric pH	9.18	Basic
Net charge at pH 7	2.1	-

#### Secondary and tertiary structure prediction of the constructed vaccine

Using the SOPMA method, the predicted secondary structure of the Vac1 was determined. The analysis revealed that 71.43% of the 21 amino acids were arranged in the alpha helix, 19.05% in the extended strand, and 4.76% in the random coil. The 3D structure of the Vac1 was predicted using PEP-FOLD3, and model 1 with the lowest energy conformations was chosen.

#### Quality assessment of predicted 3D structure

**Fig 2:**
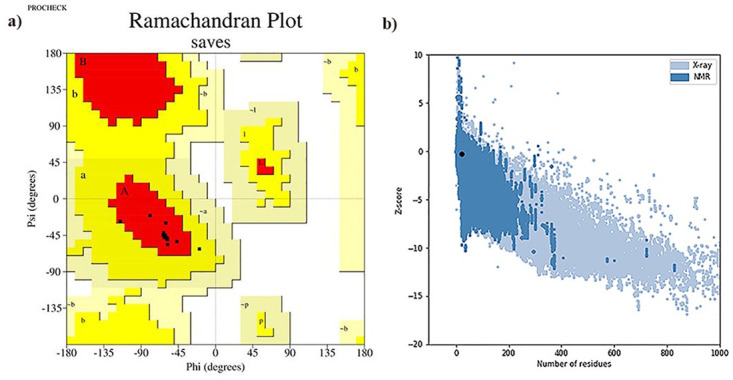
**a) Ramachandran plot of the predicted 3D structure** showing the dihedral angles Psi and Phi of amino acid residues, in which residues lie in most favored regions are in red curves (ABL) and additional allowed regions are [a,b,l,p] in dark yellow curves. **b) Z plot analysis of the predicted model.** The dark black spot represents the Z-score of the predicted model, which falls within the range of Z-scores for native protein conformations. The Z-Score plot includes Z-scores for all protein chains in the PDB that were determined through NMR spectroscopy (dark blue) and X-ray crystallography (light blue).

PROCHECK was utilized to generate a Ramachandran plot of the predicted 3D structure. The analysis revealed that 94.7% of the residues were located in the favorable region, 5.3% in the allowed region, and none in the disallowed region (Fig 2a). ERRAT computed an overall quality factor of 100%, indicating a high-resolution and well-predicted structure. Additionally, the ProSA-web tool produced a Z-score of −0.27, located within the range of native proteins of comparable size (Fig 2b). These results indicate that the predicted model is of good quality.

#### Identification of the conformational B cell epitopes

**Fig 3:**
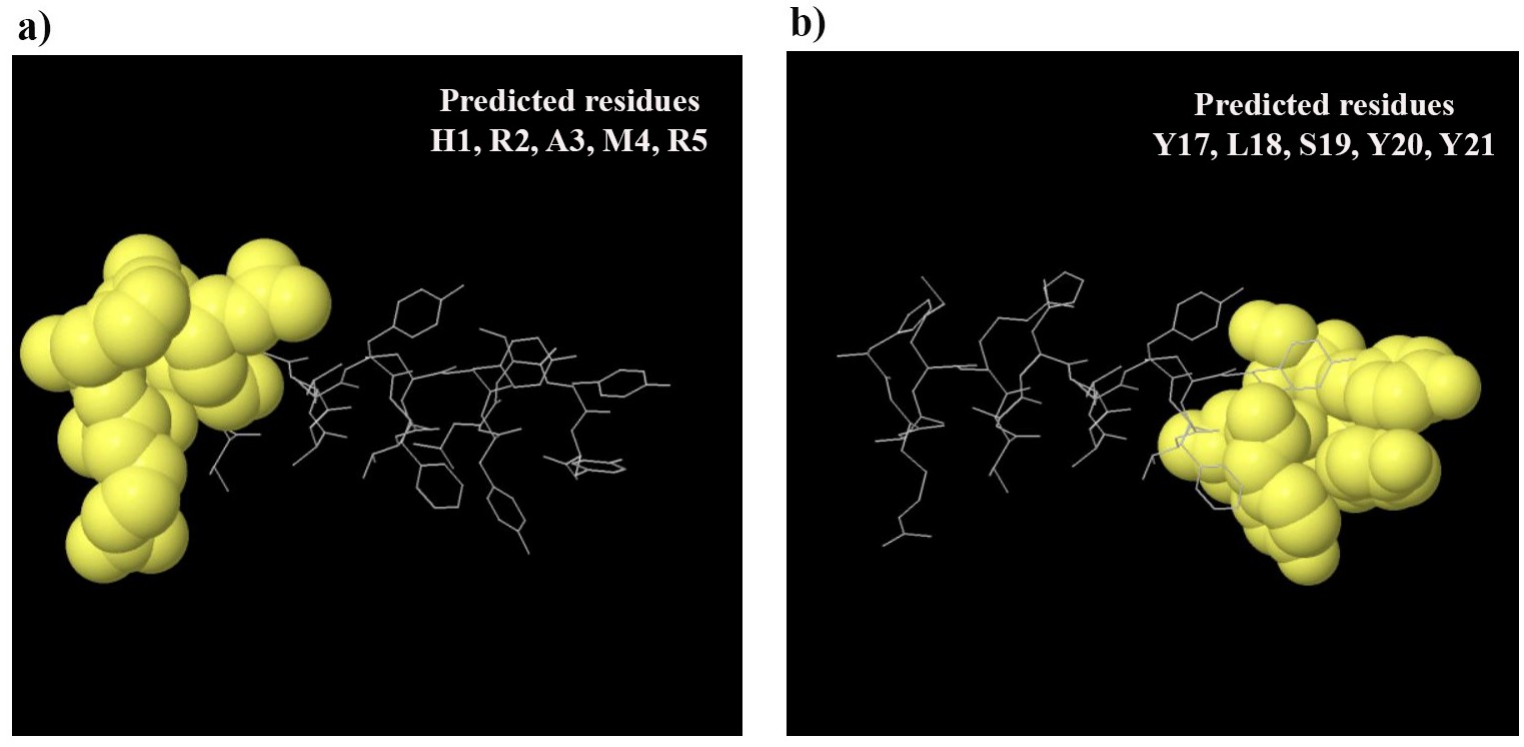
Conformational B-cell epitopes predicted using the ElliPro tool. **a)** A 3D representation of the predicted discontinuous residues in the final construct was shown as a ball-and-stick model with a score of 0.676. **b)** The ball-and-stick model depicting the predicted conformational residues in the final construct was presented in a 3D representation, with a score of 0.733. The residues corresponding to the B-cell epitopes were represented by yellow balls, while the non-epitope and core residues were depicted using white sticks.

According to the ElliPro tool results, two conformational B-cell epitopes were predicted for the constructed vaccine: (H1, R2, A3, M4, R5) with a score of 0.676, and (Y17, L18, S19, Y20, Y21) with a score of 0.733 (Fig 3).

#### Molecular docking study

HPEPDOCK 2.0 considers the binding energy between the peptide and the receptor. The result of HPEPDOCK 2.0 provided an interactive view of the top 10 models through the Jmol software, along with a summary of the docking scores and rankings for each model. The results indicated that the constructed vaccine had the highest docking energy score for the *H-2Kd* allele compared to other mouse MHC-I molecules, leading to its selection for molecular dynamics simulation. Moreover, it achieved a high docking energy score with *H-2-IAd* (mouse MHC-II molecule), as indicated in Table 6.

**Table 6:** Docking energy scores of the constructed vaccine with mouse MHC molecules (*H-2-Kd*, *H-2-Ld*, *H-2-Dd*, and *H-2-IAd*) in HPEPDOCK 2.0.

Mouse MHC molecules		Docking energy score (kcal/mol)
**MHC-I**	*H-2-Kd*	-309.906
	*H-2-Ld*	-293.336
	*H-2-Dd*	-271.828
**MHC-II**	*H-2-IAd*	-304.653

In addition, the constructed vaccine showed effective binding with both human MHC-I and MHC-II molecules. The comparison of the top 10 models of docking complex for the constructed vaccine and HLA-A*02:*01* revealed that binding model 1 has the most potent interaction energy (the most negative) with the lowest docking energy score of -367.888 (kcal/mol). The best-docked model for the constructed vaccine-HLA-DRB1*01:*01* complex exhibited a docking score of -343.313 (kcal/mol).

#### Molecular Dynamics Simulation analysis

To evaluate the stability of the docked MHC-Vaccine complexes within a dynamic environment and the effect of vaccine binding on the structure of MHC molecules, various analyses were performed on the molecular dynamics trajectories outlined below.

#### Structural analysis

**Fig 4:**
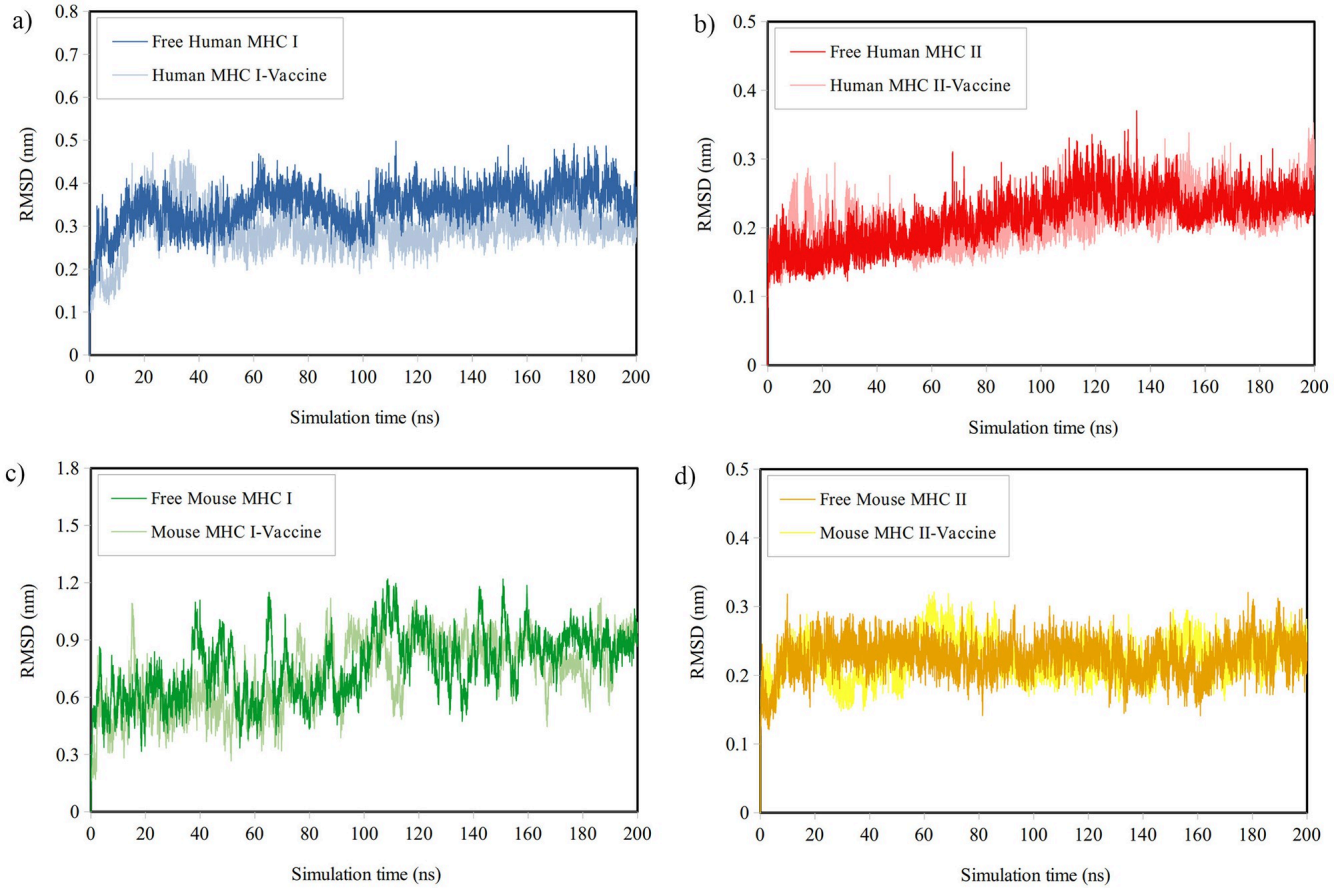
The RMSD plots for backbone atoms of a) Human MHC-I, b) Human MHC-II, c) Mouse MHC-I, and d) Mouse MHC-II both in their free form and in the presence of the vaccine during the simulation time.

*Analysis of RMSD*. Fig 4 illustrates the RMSD plots of the backbone atoms of MHCs in the presence of vaccine compared to their free form. Toward the end of the simulation, it was observed that most systems, except for Mouse MHC-I, reached equilibrium with minimal fluctuations. Table 7 provides the average RMSD, RMSF, and Rg values for free and complex MHCs during the last 50 ns, indicating the equilibrated state. Except for Mouse MHC-I, the RMSD of the backbone atoms showed limited changes throughout the simulation. Additionally, the average RMSD for Human MHC II-Vaccine and Mouse MHC II-Vaccine complexes did not significantly differ from that of free MHCs. However, the average RMSD for Human MHC I-Vaccine and Mouse MHC I-Vaccine complexes was slightly lower, showing that vaccine binding increased the stability of MHCs.

**Table 7:** Average and standard deviations of the RMSD, R_g_, and RMSF for free and complex systems during the last 50 ns.

Systems	Mean RMSD (nm)	Mean Rg (nm)	Mean RMSF (nm)
Free Human MHC-I	0.379±0.034	2.399±0.016	0.151±0.059
Human MHC I-Vaccine	0.310±0.023	2.350±0.015	0.125±0.054
Free Human MHC-II	0.235±.017	2.420±0.013	0.119±0.048
Human MHC II-Vaccine	0.238±0.026	2.439±0.011	0.135±0.067
Free Mouse MHC-I	0.876±0.089	2.260±0.120	0.293±0.117
Mouse MHC I-Vaccine	0.816±0.111	2.393±0.133	0.350±0.136
Free Mouse MHC-II	0.226±0.027	2.405±0.011	0.124±0.60
Mouse MHC II-Vaccine	0.233±0.021	2.401±0.011	0.116±0.057

**Fig 5:**
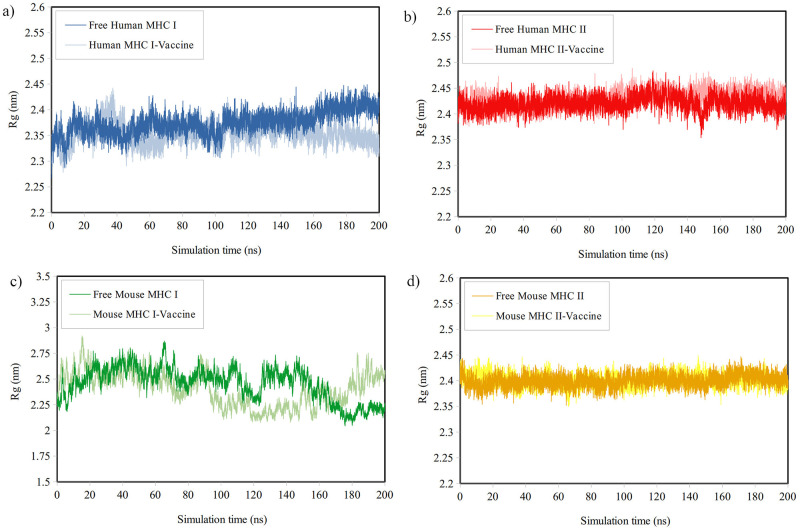
The Rg plots for a) Human MHC-I, b) Human MHC-II, c) Mouse MHC-I, and d) Mouse MHC-II both in their free form and in the presence of the vaccine throughout the simulation period.

*Analysis of R*_*g*_. Throughout the MDS, the compactness of the MHCs was evaluated by examining their Rg. Fig 5 illustrates that the Rg values for Human MHC-II and Mouse MHC-II remained relatively stable during the entire simulation. In contrast, the Rg graphs for Human MHC-I and Mouse MHC-I exhibited more fluctuations. Data analysis in Table 7 showed that the average Rg values for the Human MHC-II and Mouse MHC-I complexes with the vaccine were slightly higher than that of the free MHCs in the last 50 ns, leading to a slight decrease in compactness. Conversely, binding the vaccine to Human MHC-I and Mouse MHC-II resulted in a slight increase in compactness.

**Fig 6:**
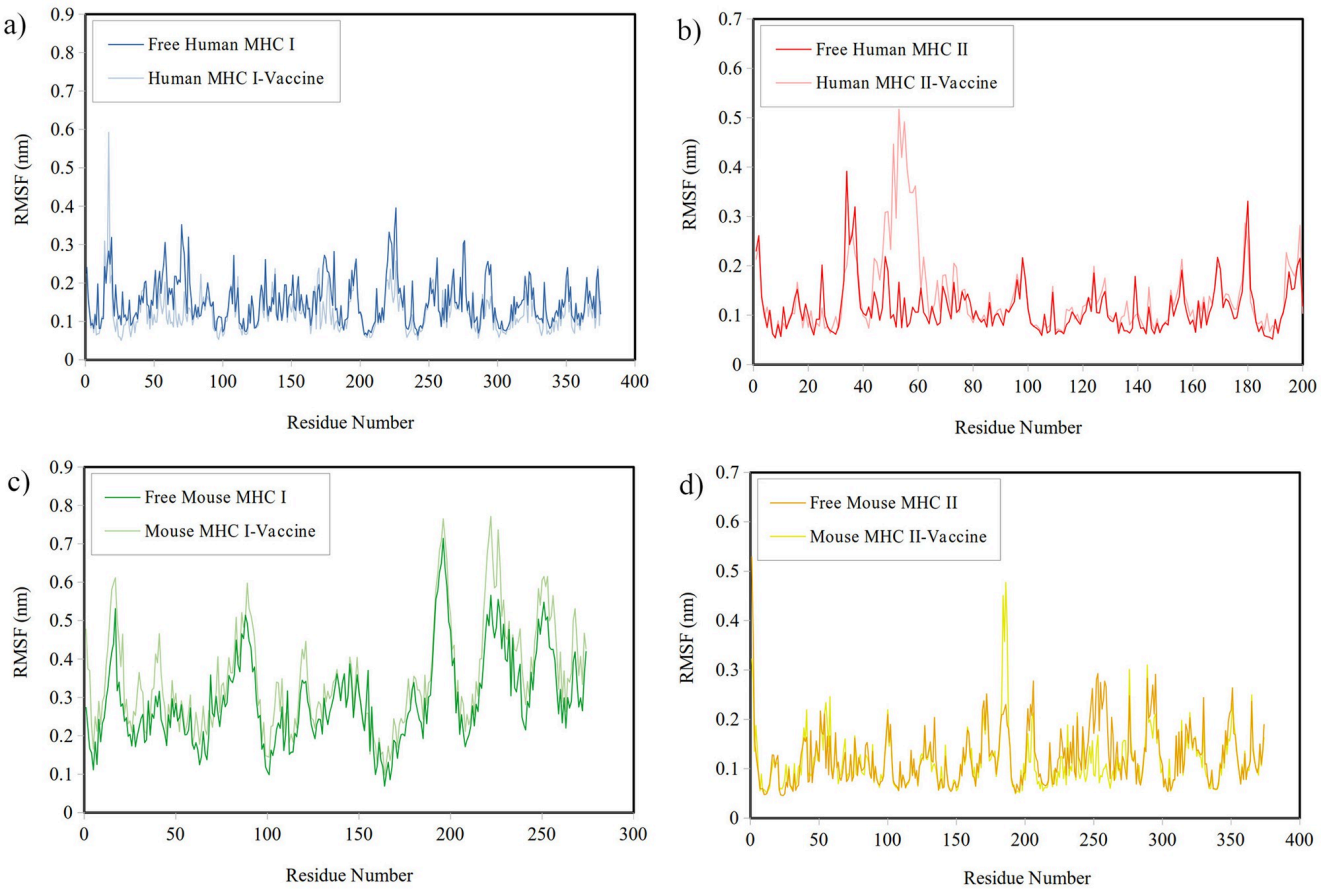
The RMSF plots for a) Human MHC-I, b) Human MHC-II, c) Mouse MHC-I, and d) Mouse MHC-II both in their free form and in the presence of the vaccine.

*Analysis of RMSF*. The local flexibility of the MHCs was examined by analyzing their RMSF in the presence and absence of the vaccine. In Fig 6, the RMSF plots for all MHCs, except for Mouse MHC-I, consistently displays low values below 0.6 nm across all regions for both the bound and unbound forms. Table 7 reveals that the mean RMSF during the last 50ns is slightly lower in the Human MHC-I and Mouse MHC-II systems for the bound MHCs than the unbound MHCs, with some exceptions showing higher fluctuations in certain regions. Conversely, the bound Human MHC-II and Mouse MHC-I systems displays an increase in mean RMSF compared to the unbound MHCs. Overall, these results indicates that the vaccine has some effect on the flexibility of the MHCs.

**Fig 7:**
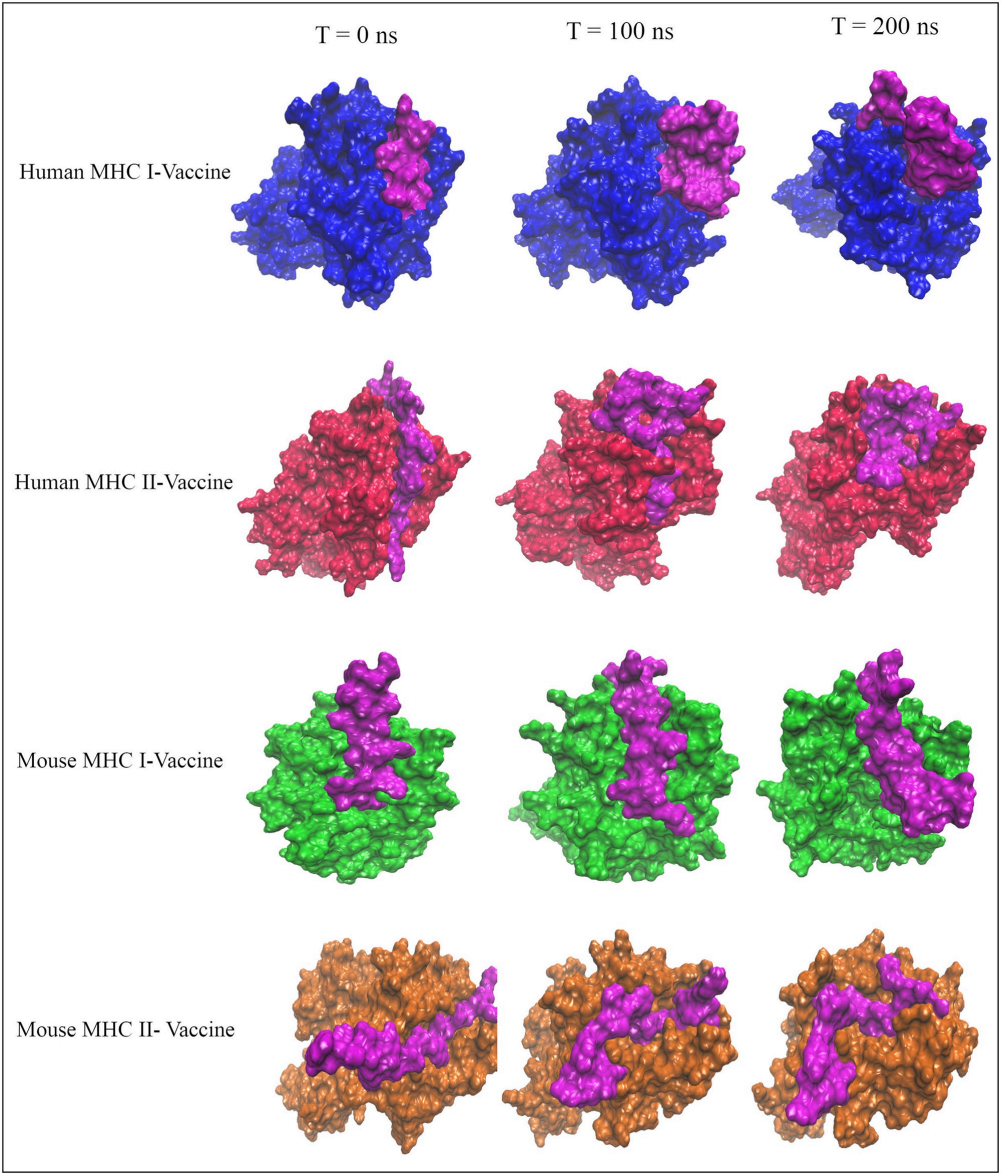
Snapshots of the MHC-vaccine systems captured every 100 ns during the simulation time. The vaccine is highlighted in magenta.

*Analysis of the snapshots*. Snapshots were captured every 100 nanoseconds during the MDS to observe alterations in the MHC-vaccine complexes. As shown in Fig 7, the vaccine consistently maintained its binding to the MHC complexes, despite variations in its position and conformation, indicating that the initial binding poses from the docking process were relatively stable. Furthermore, significant changes in the structure of the MHCs were observed throughout the simulation.

#### Interaction analysis

**Fig 8:**
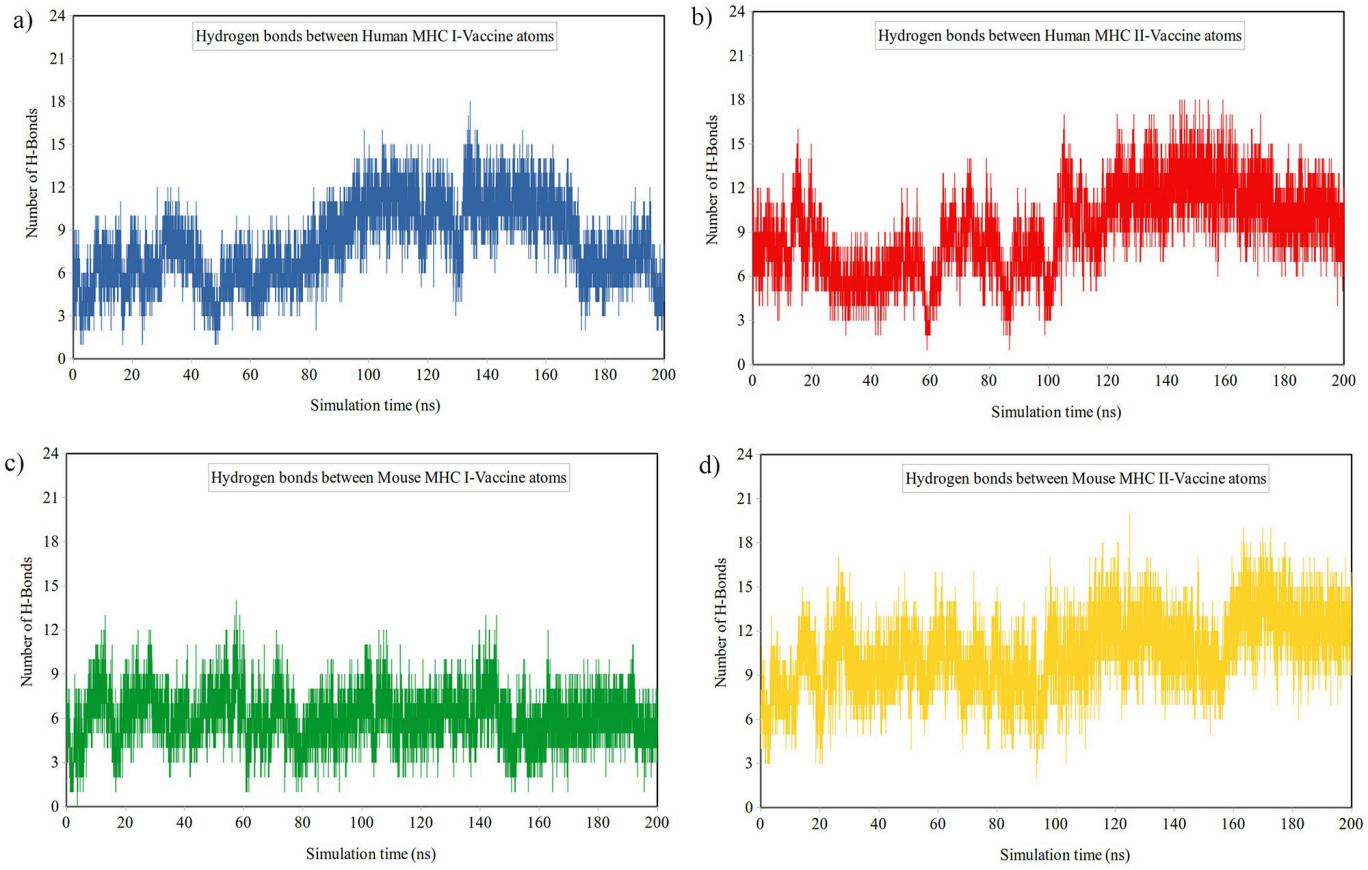
Time dependence of the number of hydrogen bonds between vaccine and a) Human MHC-I, b) Human MHC-II, c) Mouse MHC-I, and d) Mouse MHC-II during the simulation time.

*Analysis of the hydrogen bonds*. The number of hydrogen bonds, both within and between molecules, was analyzed to assess binding forces in interactions. Fig 8 displays the formation of stable hydrogen bonds between the vaccine and various MHC molecules (Human MHC-I, Human MHC-II, Mouse MHC-I, and Mouse MHC-II) during the simulation, with hydrogen bond counts reaching up to 18, 18, 14, and 20, respectively. Additionally, the average number of hydrogen bonds between MHC-MHC and MHC-solvent in the last 50 ns of the simulation was calculated and presented in Table 8. The data suggests that the presence of the vaccine led to an increase in MHC-MHC hydrogen bonds and a decrease in MHC-solvent hydrogen bonds across all MHCs, indicating the binding of the vaccine to the MHCs.

**Table 8:** Average and standard deviations of MHC-MHC and MHC-solvent hydrogen bonds during the last 50 ns.

Systems	MHC-MHC	MHC-solvent
Free Human MHC I	306.707±8.957	884.117±18.530
Human MHC I-Vaccine	312.253±9.086	878.228±17.333
Free Human MHC II	292.641±7.972	819.856±18.834
Human MHC II-Vaccine	294.610±8.443	813.530±17.625
Free Mouse MHC I	228.508±7.427	621.821±15.426
Mouse MHC I-Vaccine	233.457±7.859	607.567±16.934
Free Mouse MHC II	306.335±8.218	824.106±18.141
Mouse MHC II-Vaccine	307.766±8.523	815.061±16.432

*Analysis of MM-PBSA method*. The binding energies were examined in more depth by studying Van der Waals, electrostatic, polar solvation, and SASA energies, utilizing the MM-PBSA technique. Analysis of 1000 frames from the last 50 ns of trajectories provided insight into the key factors influencing MHC-Vaccine interactions. Results from Table 9 indicated favorable energies for Van der Waals, electrostatic, and SASA interactions, highlighting their importance in the binding mechanism. Interestingly, polar solvation energies were consistently unfavorable across the complexes. The results demonstrated that electrostatic energy played a more prominent role in vaccine binding with three MHC molecules (Human MHC-I, Human MHC-II, and Mouse MHC-II), while Van der Waals energy was more influential in vaccine binding with Mouse MHC-I. Furthermore, the hierarchy of binding energy between MHC molecules and the vaccine was determined as Human MHC-II < Mouse MHC-II < Human MHC-I < Mouse MHC-I, indicating that the binding strength between the vaccine and MHC molecules decreases in the order listed, with Human MHC-II exhibiting the strongest binding and Mouse MHC-I showing the weakest binding.

**Table 9:** Average and standard deviations of energy components for complexes analyzed by MM-PBSA.

Energy components (kJ/mol)	Human MHC I-Vaccine	Human MHC II-Vaccine	Mouse MHC I-Vaccine	Mouse MHC II-Vaccine
Van der Waals energy	-287.840 ± 26.362	-410.606 ± 24.521	-378.979 ± 21.134	-484.852 ± 29.783
Electrostatic energy	-684.001 ± 84.646	-1370.749 ± 45.663	-217.913 ± 46.686	-1045.098 ± 47.797
Polar solvation energy	713.322 ± 106.371	979.398 ± 47.211	485.766 ± 65.778	830.525 ± 69.164
SASA energy	-36.339 ± 2.803	-53.194 ± 2.172	-44.140 ± 2.275	-58.838 ± 2.990
Binding energy	-294.858 ± 58.492	-855.151 ± 42.476	-155.266 ± 61.407	-758.264 ± 64.531

**Fig 9:**
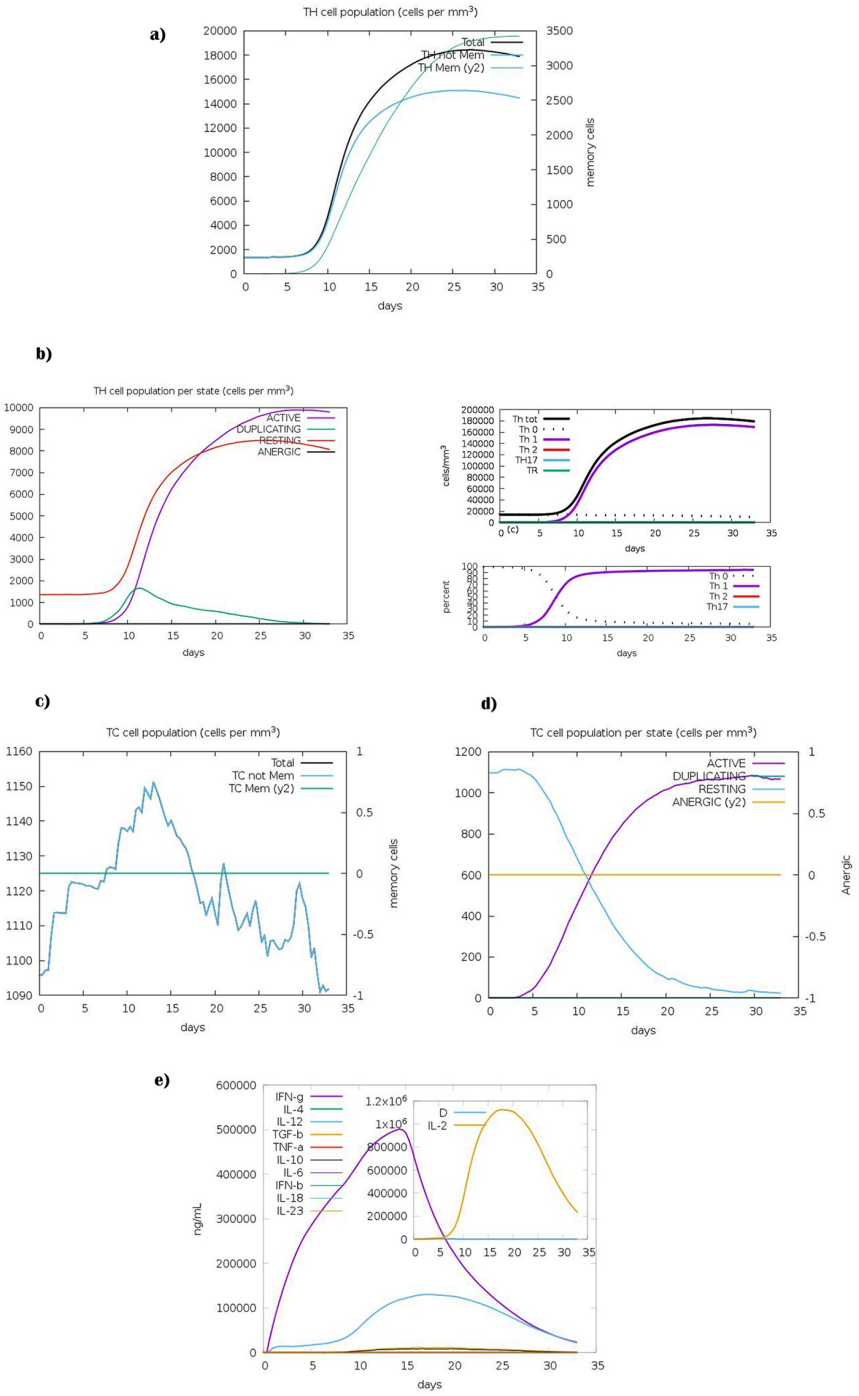
*In silico* simulation of immune response using final construct as an antigen. **a)** Helper T-cell (TH) population counts. **b)** Helper T-cell population at different states. **c)** Cytotoxic T-cell population counts. **d)** Cytotoxic T-cell population at different states. The cells not exposed to antigens are labeled as RESTING, while ANERGIC indicates the tolerance level of antigen. **e)** The cytokine profile displays an increase in IFN-γ levels following vaccine administration. Additionally, the inset graph displays levels of IL-2.

*Immune simulations of the final construct*. The simulation result using C-ImmSim revealed the development of immune response after immunization. The T helper cells, especially the Th1 cell population was highly stimulated upon immunization (Fig 9a and 9b). Similarly, the cytotoxic T-cell population (Fig 9c and 9d) was considerably increased. During the exposure time, it was also observed that levels of IFN-γ and IL-2 were also increased after immunization (Fig 9e).

### In vivo studies

#### Peptide vaccine-specific serum levels of total IgG determination by ELISA

**Fig 10:**
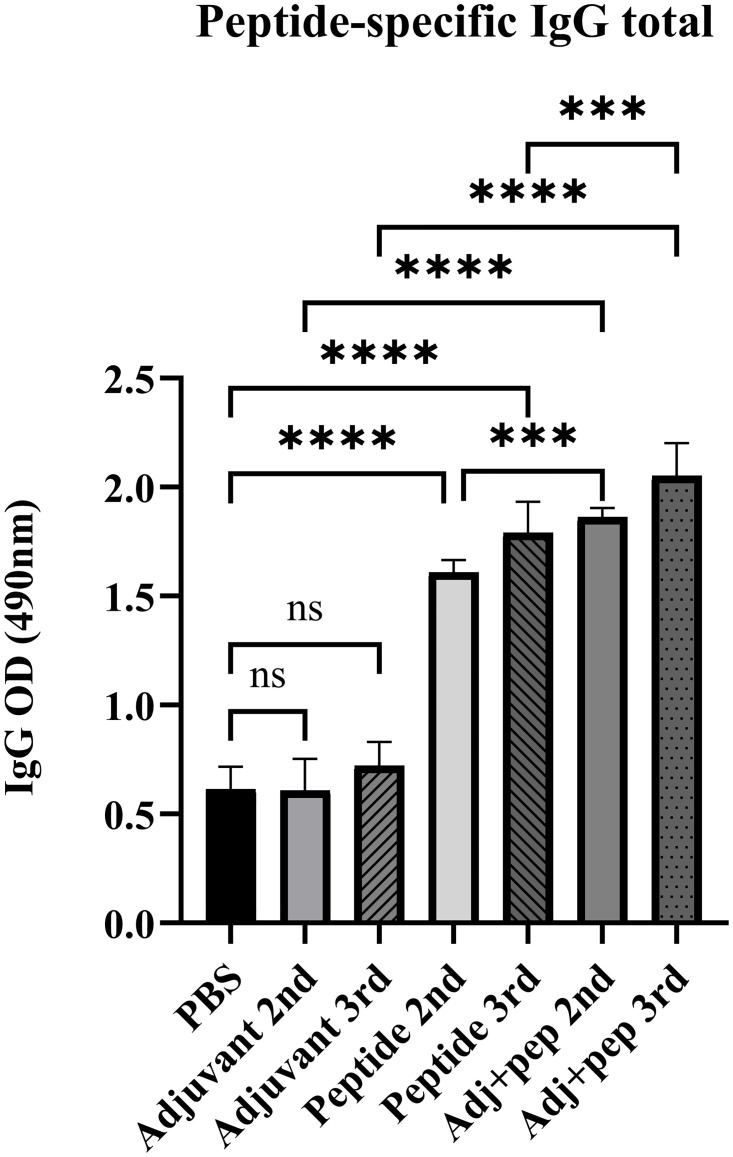
Serum levels of the peptide-specific total IgG at various groups 2 weeks after the second and third injections. The peptide was significantly capable of provoking a specific IgG response after the second and third injections without adjuvant. However, the highest level of IgG response has been achieved by Adj+pep 3rd group (ns: not significant, ***: *P* < 0.001, ****: *P* < 0.0001).

Serum levels of total IgG were significantly higher (*P* < 0.0001) in the Peptide 2nd, Peptide 3rd, Adj+pep 2nd, and Adj+pep 3rd immunization groups compared to PBS. No statistically significant difference was observed between the Adjuvant groups (Adjuvant 2nd, Adjuvant 3rd) and PBS (*P* > 0.05). In addition, the total IgG levels of mice immunized with both adjuvant and peptide in the second and third immunizations were significantly higher than those of mice immunized with peptide alone in the second and third immunizations (*P* = 0.0003 and *P* = 0.0002, respectively). Furthermore, the levels of total IgG in the Adj+pep groups (Adj+pep 2nd and Adj+pep 3rd) were much higher compared to the Adjuvant groups, with the difference being statistically significant (*P* < 0.0001). The animals that received the Adj+pep vaccine showed the highest level of IgG antibodies in their serum after the third injection (Fig 10).

#### Cytokines production

**Fig 11:**
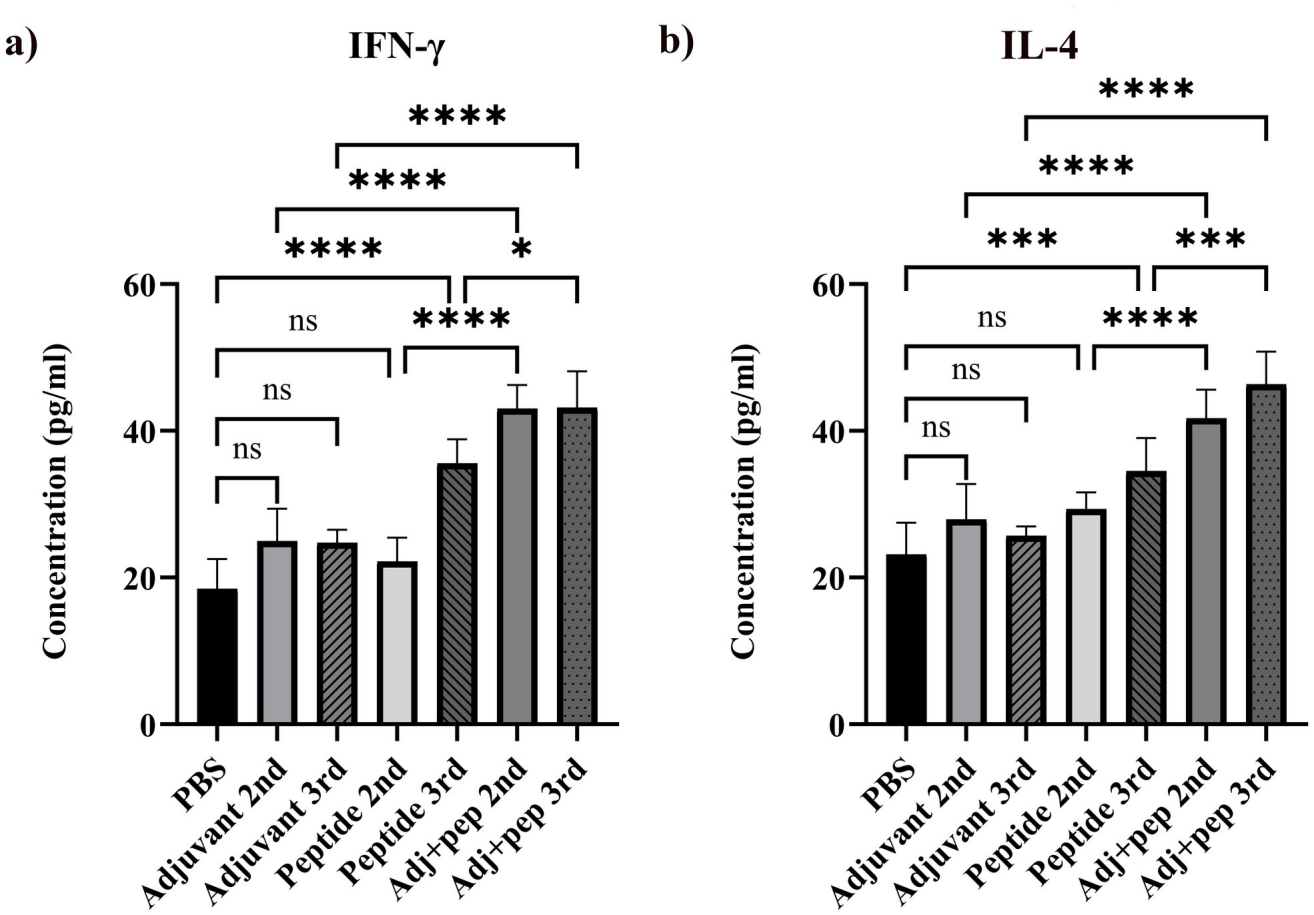
Cytokines’ levels in the supernatant of the cultured splenocytes of the immunized mice after stimulation with the peptide *in vitro*. The supernatant was collected after 48 hours of culture, and released IFN-γ and IL-4 were measured by ELISA. The peptide had a notable ability to elicit IFN-γ and IL-4 production after the third injection even without the adjuvant. However, administering the peptide with an adjuvant has resulted in higher levels of IFN-γ and IL-4. **a)** IFN-γ. **b)** IL-4 (ns: not significant, *: *P* < 0.05, ***: *P* < 0.001, ****: *P* < 0.0001).

The Adj+pep groups demonstrated a very significant difference (*P* < 0.0001) in IFN-γ production when compared to PBS. Although the splenocytes of Peptide 3rd exhibited significantly higher levels of IFN-γ compared to PBS (*P* < 0.0001), the levels of IFN-γ in the splenocytes of the Peptide immunized group after the second injection were not significantly different from the control group. No statistically significant difference was observed between the Adjuvant groups and PBS (*P* > 0.05). There was a relatively small but statistically significant difference *(P* = 0.0166) in IFN-γ production between the Adj+pep 3rd and Peptide 3rd groups. Additionally, there was a significant difference (*P* < 0.0001) in IFN-γ production between Adj+pep 2nd and Peptide 2nd. The IFN-γ production was significantly higher in the Adj+pep groups compared to the Adjuvant groups (Fig 11a).

The splenocytes of the Adj+pep immunized groups exhibited significantly (*P* < 0.0001) very high amounts of IL-4 compared to PBS. The splenocytes of Peptide 3rd exhibited considerably higher levels of IL-4 compared to PBS (*P* = 0.0002), but the splenocytes of Peptide 2nd immunized mice did not. There was no notable difference between the groups receiving adjuvants and PBS (*P* > 0.05). A highly significant difference (*P* < 0.0001) was observed in IL-4 production between Adj+pep 2nd and Peptide 2nd. The splenocytes of Adj+pep 3rd showed significantly higher levels of IL-4 compared to Peptide 3rd. The IL-4 production was significantly higher in the Adj+pep immunized groups (*P* < 0.0001) compared to the groups receiving adjuvants. The Adj+pep 3rd immunized animals’ splenocytes could secret the highest level of IL-4 after stimulation (Fig 11b).

#### Granzyme B secretion

**Fig 12:**
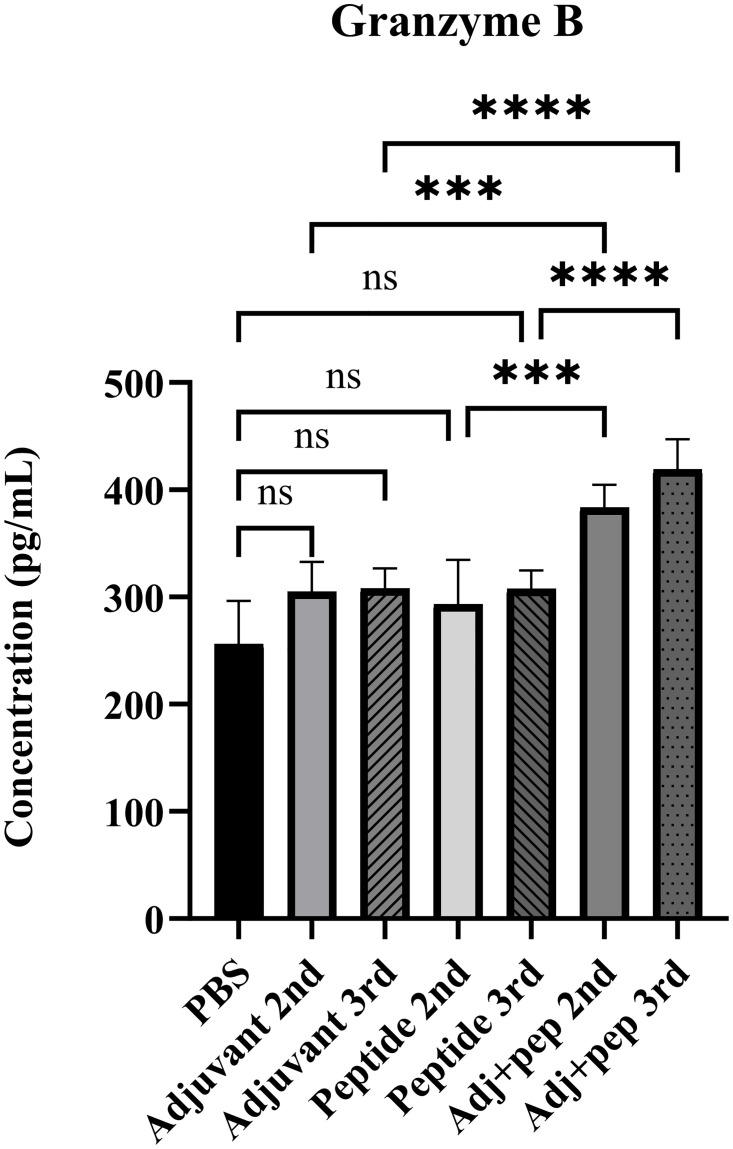
Granzyme B levels in the supernatant of the cultured splenocytes of the immunized mice after stimulation with the peptide *in vitro*. The supernatant was collected after 48 hours of culture, and granzyme B levels were measured using ELISA. After the second injection, the peptide in combination with the adjuvant demonstrated a significant ability to induce granzyme B secretion. Furthermore, the Adj+pep 3rd group showed the highest level of granzyme B following the third injection (ns: not significant, ***: *P* < 0.001, ****: *P* < 0.0001).

The results indicated that the Adj+pep groups exhibited a very significant difference (*P* < 0.0001) in granzyme B secretion compared to PBS. There was no significant difference between the Adjuvant groups and PBS, or between the Peptide groups and PBS (*P* > 0.05). The Adj+pep 2nd group had higher levels of granzyme B production compared to Peptide 2nd, with a *P* value of 0.0001, and Adjuvant 2nd, with a *P* value of 0.0008. The immunizing animals with Adj+pep after the third injection resulted in significantly elevated levels of granzyme B production (*P* < 0.0001) compared to the Peptide 3rd and Adjuvant 3rd groups. The spleen cells of animals immunized with the Adj+pep 3rd vaccine produced the highest amount of granzyme B upon stimulation (Fig 12).

#### Lymphocyte proliferation

**Fig 13:**
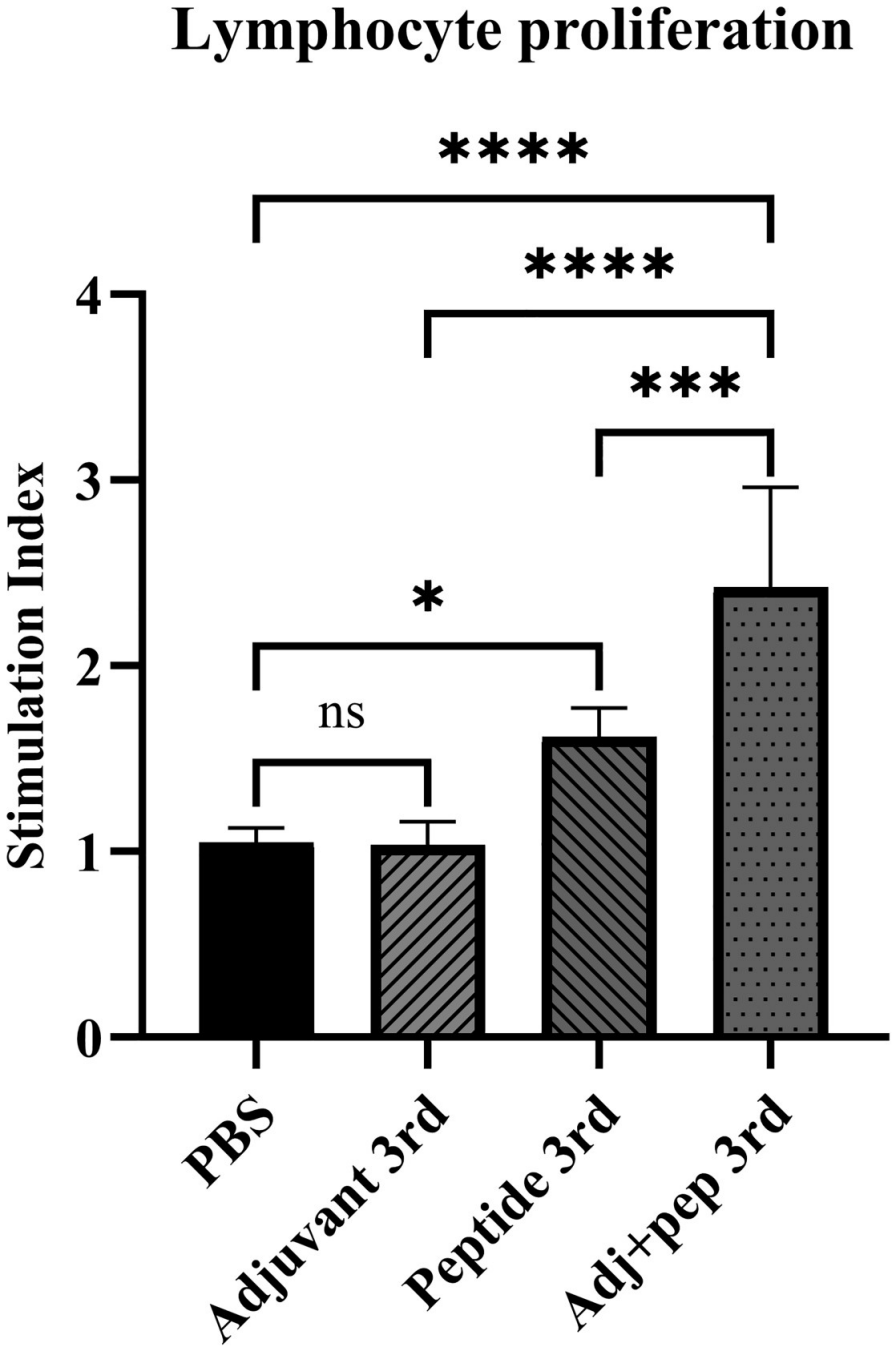
The proliferation rates of mouse lymphocyte cells (MTT assay) after 72 hours of exposure to peptide expressed as stimulation index. The stimulation index (SI) corresponds to antigen-stimulated wells’ OD/ unstimulated wells’ OD. The peptide resulted in a notable proliferation rate of mouse lymphocyte cells after the third injection, even without an adjuvant. However, the highest level of stimulation index has been observed when the peptide is administered along with an adjuvant after the third injection (ns: not significant, *: *P* < 0.05, ***: *P* < 0.001, ****: *P* < 0.0001).

As Fig 13 illustrates, the Adj+pep 3rd immunized mice exhibited the highest lymphocyte proliferation rate expressed as stimulation index in comparison with other groups. They showed a very significant difference (*P* < 0.0001) with PBS and Adjuvant 3rd groups. Compared to PBS, Adjuvant 3rd did not show any significant difference. Peptide 3rd vaccination caused higher lymphocyte proliferation (*P* = 0.0138) than PBS, and a significant difference (*P* = 0.0006) was observed between this group and Adj+pep 3rd.

#### Vaccine safety

There were no observed changes in the appearance, behavioral patterns, food intake, and weight loss of the mice.

## Discussion

The epitope-based vaccine is gaining more attention in combating infectious agents, including parasites, bacteria, fungi, viruses, and even cancers [48]. Computational epitope-based vaccine design emerges as a cost-effective and time-efficient approach. This methodology leads to creating vaccines with maximal therapeutic efficacy and minimal adverse reactions [49]. In 2023, bioinformatics methods were utilized to develop epitope-based vaccines for langya henipavirus (LayV) and severe acute respiratory syndrome coronavirus 2 (SARS-CoV-2). These vaccines demonstrated favorable physicochemical characteristics, including antigenicity, safety, stability, and inducing both humoral and cellular immune responses [50, 51]. Additionally, Ullah *et al*. and Malik *et al*. designed *in silico* epitope-based vaccines targeting *Proteus penneri* and *Brucella Melitensis* pathogens. These vaccines exhibited high binding affinity with MHC-I and MHC-II molecules and displayed stable dynamics with notable Van der Waals and electrostatic energies [52, 53].

The efficacy of a cancer vaccine relies on carefully selecting suitable cytotoxic T lymphocyte epitopes, T-helper epitopes, and adjuvants [54]. The use of immunodominant epitopes derived from cancer/testis antigens in anti-breast cancer vaccination has shown to be highly effective [55]. Brother of the regulator of imprinted sites (BORIS) [56], acrosin binding protein (ACRBP), synaptonemal complex protein 1 (SYCP1) [57, 58], and transmembrane protein 31 (TMEM31) [59] cancer/testis antigens have utilized for *in silico* peptide vaccine design due to their high expression in various tumors. The final constructs, comprising cytotoxic T lymphocyte epitopes and various B-cell epitopes, exhibited attributes of stability, immunogenicity, and non-allergenicity. NY-SAR-35 antigen, previously unexplored in bioinformatics, shares characteristics with BORIS, TMEM31, ACRBP, and SYCP1 in terms of its high expression on the cancer cells, notable immunogenicity, and absence of expression on the normal cells. The broad expression of NY-SAR-35 antigen across different cancer types suggests a degree of universality that makes it attractive target and excellent candidate for broad-spectrum cancer immunotherapy. Furthermore, the restricted expression of NY-SAR-35 in normal tissues minimizes the risk of autoimmune reactions or off-target effects. For these reasons, the cancer/testis antigen NY-SAR-35 was used in this study to design a peptide vaccine against breast cancer using various bioinformatics tools. Similar to the previously discussed constructs, our developed vaccine was highly antigenic and non-allergenic, indicating its ability to elicit strong immune responses without inducing any undesirable allergic reactions. It was non-toxic, stable, and IFN-γ and IL-4 inducer. Furthermore, it could bind with human and mouse MHC molecules, exhibiting high binding affinity and strong interaction. Molecular dynamics simulations were performed to further explore the interaction between the vaccine and MHCs, and to evaluate the stability of the docked MHC-Vaccine complexes. The findings confirmed the favorable stability of most complexes, highlighting sustained interactions between the MHC molecules and our constructed vaccine, and emphasizing the presence of considerable Van der Waals and electrostatic energies. The constructed vaccine had two conformational B cell epitopes that are likely to interact with B cells, responsible for producing antibodies. As a result, it is expected to stimulate a humoral immune response, which should be confirmed through experimental models. Using immune simulation *in silico*, an apparent increase in IFN-γ levels alongside the activation and proliferation of cytotoxic cells and Th1 cells was revealed. To ensure the efficacy of our designed vaccine, it was crucial to conduct *in vivo* investigations. Thus, the immunogenicity of our peptide vaccine was evaluated in BALB/c mice. Our investigation focused on examining the production of IgG, which serves as a representative of the humoral immune response, and granzyme B, which represents the cytotoxic activity of T cells. Additionally, the investigation involved measuring cytokine levels, specifically IFN-γ and IL-4. The activation of Th1 cells leads to the production of IFN-γ, which can stimulate the cellular immune response. On the other hand, the activation of Th2 cells can lead to the production of IL-4, which initiates the humoral immune response.

The *in silico*-designed vaccines have relatively low immunogenicity compared to live attenuated vaccines, which is a big drawback. To address this issue, adjuvants are often employed to enhance their immunogenicity. In this study, incomplete Freund’s adjuvant (IFA) was used. The splenocytes of immunized groups with our peptide vaccine emulsified in IFA had significantly higher levels of IFN-γ and IL-4 cytokines than PBS. Additionally, it was found to increase the serum level of specific IgG when administered to BALB/c mice. In 2021, Mahdevar *et al*. developed a peptide vaccine using the cancer/testis antigen BORIS. They reported that the splenocytes of immunized groups with the peptide vaccine emulsified in IFA exhibited significantly higher amounts of IFN-γ and IL-4 cytokines secretion than those treated with PBS. Additionally, this vaccine increased the serum level of total IgG when administered to BALB/c mice [46]. Our findings are consistent with the results obtained by the previous study. Although our peptide vaccine alone was able to elicit some immune responses, the addition of IFA to the peptide resulted in more significant increases in immune cell activation. Specifically, the emulsification of the peptide with the adjuvant led to higher levels of the cytokines IFN-γ and IL-4, indicating the activation of both Th1 and Th2 cells. Adjuvants work by creating a deposit of the antigenic compound at the vaccine site, which is gradually released over time, thereby extending the duration of the immune response. The gradual release of the oil-deposited antigen over time, facilitated by the emulsification process, resulted in a more sustained presence of the antigen and prolonged exposure to it, leading to a more robust immune response. Overall, the cytokine induction profile suggested both cellular and humoral immune responses. IFN-γ has various effects on mature B-cells, including the induction of IgG production [60]. According to that, the administration of the peptide alone led to the production of IFN-γ, consequently inducing IgG production. A significant increase in IgG levels was observed compared to a control group receiving PBS. However, when the peptide was combined with IFA, there was a more significant difference in IgG antibody levels between the two groups, indicating that the adjuvant enhanced the vaccine’s effectiveness in stimulating an immune response.

In 2020, Safavi *et al*. created a peptide vaccine that included SYCP1 and ACRBP epitopes. Upon immunization of mice with the vaccine emulsified in IFA, the researchers observed an increase in granzyme B production from isolated splenocytes compared to the control [47]. In our study, Adj+pep groups exhibited a notable elevation in granzyme B levels. This result agreed with the findings of the previous research. The cytokine IFN-γ, produced by immune cells, affects the behavior of various immune cells within the tumor microenvironment. It plays a crucial role in stimulating the immune system’s anti-cancer defenses by boosting the activity of specific immune cells, including CD4+ T helper cells, CD8+ cytotoxic T cells, natural killer cells, dendritic cells, and macrophages. Additionally, it promotes the presentation of antigens to these immune cells, which helps to trigger an effective immune response against cancer cells [61]. In the current study, the observed elevation in granzyme B levels indicates the activation of cytotoxic cells, specifically cytotoxic T lymphocytes and natural killer (NK) cells. This increase can be attributed to the administration of the combination of adjuvant and peptide (Adj+pep), which induced IFN-γ production, consequently enhancing the activity of cytotoxic T cells and natural killer cells. These immune cells play a pivotal role in cellular immunity, particularly in combating tumor cells that express tumor antigens. When a cytotoxic T cell or NK cell identifies a target cell, granzyme B is released and enters the target cell. Once inside, granzyme B initiates a process that leads to programmed cell death, or apoptosis, in the target cell.

As a result of all the aforementioned outcomes, the Adj+pep groups in our study demonstrated the most potent immune response in both the humoral and cellular arms.

Increased cell proliferation following vaccination is known as a higher stimulation index. As observed in the studies of Mahdevar *et al*. and Safavi *et al*., the peptide vaccines emulsified in IFA resulted in higher lymphocyte proliferation compared to PBS [46, 47]. Our study yielded similar results, demonstrating that our peptide vaccine, whether administered alone or emulsified in the adjuvant, could stimulate and promote the proliferation of mouse splenocytes compared to those not exposed to the peptide. Although the peptide alone stimulated the proliferation of mouse lymphocytes, there was a significant difference in the stimulation of lymphocyte proliferation between the peptide group and the group that received the peptide emulsified in the adjuvant. This finding confirms the crucial role of adjuvants in optimizing the performance of vaccines.

Upon comparing experimental results with those generated through bioinformatics, a notable match between them was observed, emphasizing the power of bioinformatics tools in predicting and developing peptide vaccines and reinforcing the importance of bioinformatics in the field of vaccine design.

Our peptide vaccine presents several advantages. It is cost-effective, safe, easy to design, and simple to produce. It can be conveniently administered via subcutaneous injection. Furthermore, it can exhibit notable efficacy by inducing both humoral and cellular immune responses. Despite these advantages, it has one notable disadvantage: its therapeutic effectiveness is hampered by tumor heterogeneity and the potential for immune evasion. Tumor heterogeneity refers to various subclones within the tumor, each with distinct properties and antigen expression patterns. Targeting a single antigen like NY-SAR-35 may only affect a subset of these tumor subclones. Additionally, cancer cells may evade immune recognition through antigen editing or poor antigen presentation, potentially concealing the targeted antigen. To overcome this matter, numerous studies have utilized diverse sources of tumor-associated antigens for anti-cancer vaccination. In the study of Safavi *et al*., testicular germ cells and sperm cells, well-known sources of CTAs, were investigated for anti-breast cancer vaccination in BALB/c mice. These vaccinations demonstrated potent therapeutic effects [62].

The designed peptide vaccine holds promise as a prophylactic vaccine for high-risk breast cancer patients. Despite concerns about the suboptimal effectiveness of immunotherapy regimens in treating advanced cancer patients [63], numerous studies suggest that cancer vaccines can inhibit tumor growth in high-risk individuals, such as those exposed to occupational carcinogens [64] or bearing hereditary mutations in tumor suppressor or oncogenes [65, 66]. A major challenge in developing prophylactic cancer vaccines lies in the direct transition of these vaccines from preclinical investigations to large primary prevention trials involving humans. Therefore, many studies suggest that a more practical approach would progress from successful primary cancer prevention in murine models to tertiary prevention in humans (preventing cancer in those already at risk), and only then to primary (preventing cancer from occurring in individuals who are cancer-free) or secondary prevention (early detection and treatment of pre-cancerous lesions or early-stage cancers). Cancer vaccines may offer therapeutic benefits in managing cancer recurrence and metastasis among patients with minimal residual cancerous lesions [63, 67]. Peptide vaccines show potential as a promising strategy to be designed and employed in this context [68]. Considering the time limitation for hindering the possibility of metastasis, it appears that vaccination strategies, such as immunization against NY-SAR-35 antigen in patients who exhibit high expression levels of the target antigen in the primary tumor, have promising potential as a method to pursue following an initial cancer diagnosis. To assess the anti-tumor effects of our vaccine in future animal experiments, an animal cancer model injected with a breast cancer cell line expressing the NY-SAR-35 antigen must be utilized for a more accurate evaluation and understanding of the potential effectiveness of our vaccine against this type of cancer.

## Conclusion

In this study, a novel cancer vaccine utilizing the NY-SAR-35 antigen was designed by bioinformatics tools, and its efficacy was evaluated in BALB/c mice. It was antigenic, non-allergenic, non-toxic, stable, and able to induce IFN-γ and IL-4. Moreover, *in vivo* experiments confirmed that our designed vaccine could provoke strong humoral and cell-mediated immune responses. These findings suggest that our vaccine may be a promising candidate for preventing breast cancer in high-risk populations. Despite the promising results observed in animal models, further experiments and clinical trials are required to fully demonstrate the efficacy of this peptide vaccine in various aspects.

## Supporting information

S1 File Dataset of RMSD for free and complex systems. (XLSX)

S2 File Dataset of Rg for free and complex systems. (XLSX)

S3 File Dataset of RMSF for free and complex systems. (XLSX)

S4 File Dataset of hydrogen bonds for free and complex systems. (XLSX)
